# Read-mapping signatures for molecular characterization of transgenic DNA insertions in paired-end Illumina data

**DOI:** 10.1186/s12896-026-01185-3

**Published:** 2026-06-26

**Authors:** Colton R. Kessenich, Nicole Catherall, Elisa Hughes, Kyle Skottke, Jason Vest, Kristen E. Wendt

**Affiliations:** Bayer Crop Science, 700 Chesterfield Pkwy W, Chesterfield, MO 63017 USA

**Keywords:** Whole genome sequencing, Transgenic plants, T-DNA insertion, Read mapping, Junction sequence analysis, GMO detection, Illumina sequencing, Event characterization

## Abstract

Transgenic crops undergo rigorous safety assessments prior to commercialization, with molecular characterization serving as a critical component of regulatory review. This process establishes the identity, copy number, sequence integrity, absence of unintended foreign DNA, and insert stability across breeding generations. While whole-genome sequencing (WGS) has emerged as a powerful alternative to Southern blotting, the lack of accessible interpretation frameworks can be an entry barrier to those who wish to understand this modernized experimental setup. We developed an analytical workflow based on mapped-read signatures to characterize T-DNA (transfer DNA) insertions using short-read WGS data. Simulated Illumina paired-end datasets representing diverse transformation outcomes were generated and analyzed to define five informative read classes, which when observed mapped to a reference transformation construct provide distinct signatures indicating transformation outcomes. These signatures were applied to identify insertion boundaries, copy number, structural anomalies, and potential contamination. Mapped-read signatures can reliably distinguish single-copy inserts, multiple insertions, backbone co-integrations, and structural rearrangements, aided by coverage profiles and mate-pair orientations. We present representative examples and a practical interpretation to guide practitioners new to WGS-based molecular characterization and regulators assessing these data. This framework standardizes interpretation of short-read paired-end WGS data for molecular characterization without prescribing specific software. The platform-agnostic approach ensures broad applicability while enhancing transparency in regulatory assessments.

**Supplementary Information:**

The online version contains supplementary material available at https://doi.org/10.1186/s12896-026-01185-3.

## Introduction

Molecular characterization is a critical component of regulatory safety assessments of biotechnology-derived products. It determines which segments of the transformation construct have integrated into the genome, the exact genomic location of any insertion, the number of inserted copies, the integrity and completeness of any inserted sequence, the absence of unintended plasmid sequences, and whether any insertion is stably inherited across breeding [1–3]. Historically, a combination of Southern blotting and targeted (site-directed) sequencing has been used for molecular characterization. However, Southern blotting is labor-intensive and prone to ambiguous results due to probe cross-hybridization, incomplete digestion, star activity, anomalous band mobility, or complex insertions [4, 5].

Whole genome sequencing (hereafter referred to as WGS), combined with bioinformatics, has emerged as a sensitive, efficient, and cost-effective alternative. WGS provides nucleotide-level resolution in a single standardized workflow, contrasting with the multi-step approach of Southern blotting with custom probes followed by targeted sequencing. These technical advantages, coupled with decreasing sequencing costs and technological maturation of high-throughput sequencing platforms, have driven global regulatory acceptance over the past decade [6]. Agencies in the EU (EFSA), United States, Korea (MFDS), Canada, Japan, China, Brazil, Argentina, and numerous other countries now accept WGS data in regulatory submissions [1, 6–8]. Despite the absence of formal methodological guidance, the United States was the first to accept WGS data in 2012 [6]. Canada’s 2017 guidance was the first regulatory document specifically addressing WGS-based molecular characterization of genetically modified plants [8], quickly followed by EFSA’s Technical Note (2018) establishing standards for both Sanger and high-throughput WGS sequencing approaches [9, 10]. These documents reflect a growing international consensus on the utility of WGS for molecular characterization, yet practical frameworks for data interpretation remain limited.

Despite its advantages, WGS analysis can be challenging for those unfamiliar with bioinformatics [5, 11], and a standardized molecular characterization workflow is lacking. Various WGS and targeted sequencing methods have been used successfully in molecular characterizations [12–21], each employing different bioinformatic strategies. This diversity in approaches highlights the method’s flexibility but also presents challenges for consistent interpretation by researchers and regulators. Furthermore, in both academic and industry settings, often only ideal transgenic events are advanced for research manuscripts and commercialized products. Consequently, data representing imperfect transformations are largely excluded from literature. This inadvertently creates a barrier on how to interpret off-type paired-end sequencing-based WGS characterizations for those who want to become experts in interpreting these data.

To support implementation of WGS-based molecular characterization, we present examples representing diverse transformation outcomes alongside interpretation guidance. The framework relies on characteristic mapping signatures produced by reads spanning construct-to-genome boundaries, supplemented by coverage depth and mate-pair information. All examples assume Illumina short-read paired-end sequencing.

## Materials and methods

This study uses simulated sequencing data to illustrate how to interpret results from a molecular characterization workflow. The simulation followed six steps: (1) design a transformation construct, (2) simulate random T-DNA insertion(s), (3) generate representative sequencing reads, (4) map reads to the construct, (5) visualize alignments, and (6) interpret insertion signatures. Each step is detailed below.

A 14,260 bp transformation construct and 1 Mb host “genome” were simulated using randomgenome.sh (BBTools v39.56; random seeds 28049 and 16533, respectively; all other parameters default) [22]. A representative construct map (Fig. 1) was created relative to this sequence, dividing it into two regions: the Intended T-DNA and the Construct Backbone. The overall architecture and elements (promoters, coding regions, selectable markers, etc.) were arranged to reflect typical *Agrobacterium*-mediated vectors commonly used in commercial plant transformations.

**Fig. 1:**
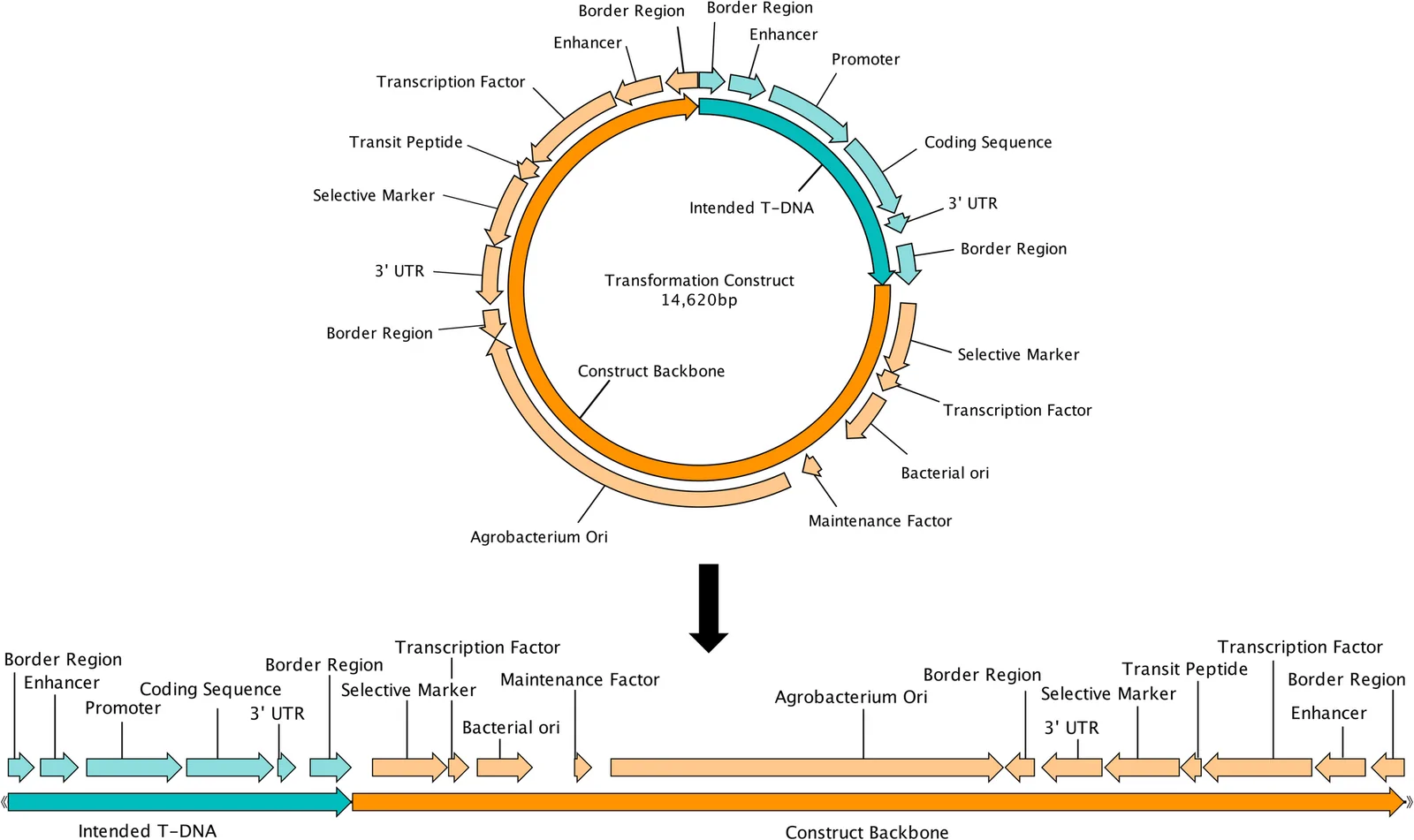
Transformation construct showing T-DNA and backbone regions in circular and linear formats. The 14,260 bp construct is divided into intended T-DNA and construct backbone regions, reflecting typical Agrobacterium-mediated vectors. Generic annotations (e.g., promoter, coding sequence) represent common genetic elements

The single transformed genome was generated by inserting construct bases 280 - 3,416 at position 500,001. For double copy insertion, a secondary insert was generated by inserting construct bases 263 - 3,194 at position 750,001 of the previously transformed sequence creating two independent insertions separated by approximately 247 kb. The 5 kb region from base 85,001 to 90,000 of the genome was selected to serve as the endogenous single copy locus (SCL) control region. The construct was used both in full (simulated control) and in subsampled forms to simulate various common transformation outcomes and observations.

Simulated Illumina data were generated for each scenario using the “randomreads.sh” script from the BBMap package v39.56 [22, 23], with the following parameters: 150 bp read length, insert sizes between 400 and 550 bp (mimicking size selection during sequencing library preparation), Gaussian distribution of insert sizes, seed value of 7, and paired-end output. By default, randomreads.sh simulates realistic Illumina errors via substitutions that increase toward read ends (higher 3’ rates), mimicking real sequencing data. These appear as sporadic colored mismatches in aligned reads during visualization (detailed in Results), distinct from the structural junction signals that define insertion boundaries. Coverage was set to 75×, except for contamination scenarios, which used 3× coverage (low-level contamination/sub-genomic equivalent). For bacterial contamination, additional parameters maxsnps = 7, snprate = 1, insrate = 0.25, delrate = 0.25, maxinss = 1, and maxdels = 1 were applied to simulate genomic divergence. All simulated datasets assume a homozygous inbred diploid plant background.

For the real-world example, genomic DNA was extracted from seed tissue of MON 89788 and its conventional parent line following a modified Rogers and Bendich [24] method coupled with Qiagen MaXtract High Density tubes to aid in phase separation and a polyethylene glycol precipitation (PEG-8000). Prior to DNA isolation, the seed were treated to remove environmental contamination using a series of Tween 20, NaOCl and HCl rinses. Paired-end libraries (size selected to ~ 500 bp insert size) were prepared from sonicated DNA (Covaris E210) using the KAPA Hyper kit with TruSeq UD Indexes, then sequenced to generate ~150 bp reads on an Illumina NovaSeq 6000 at Bayer Crop Science. These reads were aligned to the proprietary MON 89788 transformation construct (T- DNA plus plasmid backbone) used for event generation; the T-DNA portion of this construct is identical to the publicly available sequence (GenBank HW805122.1), which fully contains the T-DNA region shown in Fig. 9. No reads aligned to the backbone region of the construct. Reads were also aligned to the soybean *Le1* single-copy locus (GenBank K00821.1) [20] for empirical depth calibration.

Reads were mapped using Bowtie2 with the “very-fast-local” preset and sorted using SAMtools [25] (v2.5.4 and v1.21 for simulated data; v2.3.4.1 and v1.10 for the real-world dataset, which was generated and analyzed in 2024). The “–no-unal” flag was additionally applied to the real-world alignment to omit unaligned reads from the output. By default, Bowtie2 (like most short-read aligners) reports one alignment per read when multiple equally-optimal positions exist, selecting among tied alignments pseudo-randomly [26]. Mapping visualizations were created using Integrative Genomics Viewer (IGV) v2.16.0 [27], with reads grouped by pair orientation, visualized as squished, viewed as pairs, mismatched bases shown, and soft-clipping on. Pairwise comparisons were done using FASTA36 v36.3.8i [28, 29].

The reads, mappings, references, and any deviations from the default generation parameters for their synthesis are documented and available in the supplemental data package [30].

## Results and discussion

A straightforward approach to WGS-based characterization involves sequencing the entire genome of the transformed plant and mapping reads to the full transformation construct, including backbone sequences [12, 14, 16, 18–21], ensuring the detection of any unintended backbone integration. A typical WGS-based molecular characterization experiment requires sequencing at least three DNA samples applied in five reference-mapping combinations (Table 1).

**Table 1:** Recommended samples and reference-mapping combinations for WGS-based molecular characterization of transgenic events

Sample sequenced	Reference sequence	Purpose	Expected observations
Transformation construct	Transformation construct	Positive control: establish sequencability	Uniform coverage and/or knowledge of any non-uniformity
Transformed plant (test material)	Transformation construct	Detection and characterization of any insertions	Discrete junction pileups; coverage across insert(s) ~ consistent with SCL depth expectations
Non-transformed plant (conventional control)	Transformation construct	Negative control: detect any homology between host genome and transformation construct	No/low uniform coverage; reads only at homologous regions
Transformed plant (test material)	Single-copy locus (SCL)	Empirical verification of sequencing depth	Uniform coverage; ~ genome depth
Non-transformed plant (conventional control)	Single-copy locus (SCL)	Empirical verification of sequencing depth,	Uniform coverage; ~ genome depth

### Read classes and mapping signatures

When short-read paired-end sequencing data (e.g., Illumina) from a transgenic plant are mapped to the transformation construct, the resulting read alignments can be binned into 5 distinct categories (Fig. 2). These categories provide the interpretive basis for analyzing mapping signatures: together with coverage depth and mate-pair information, they enable characterization of T-DNA insertions demonstrated in the following examples.

**Fig. 2:**
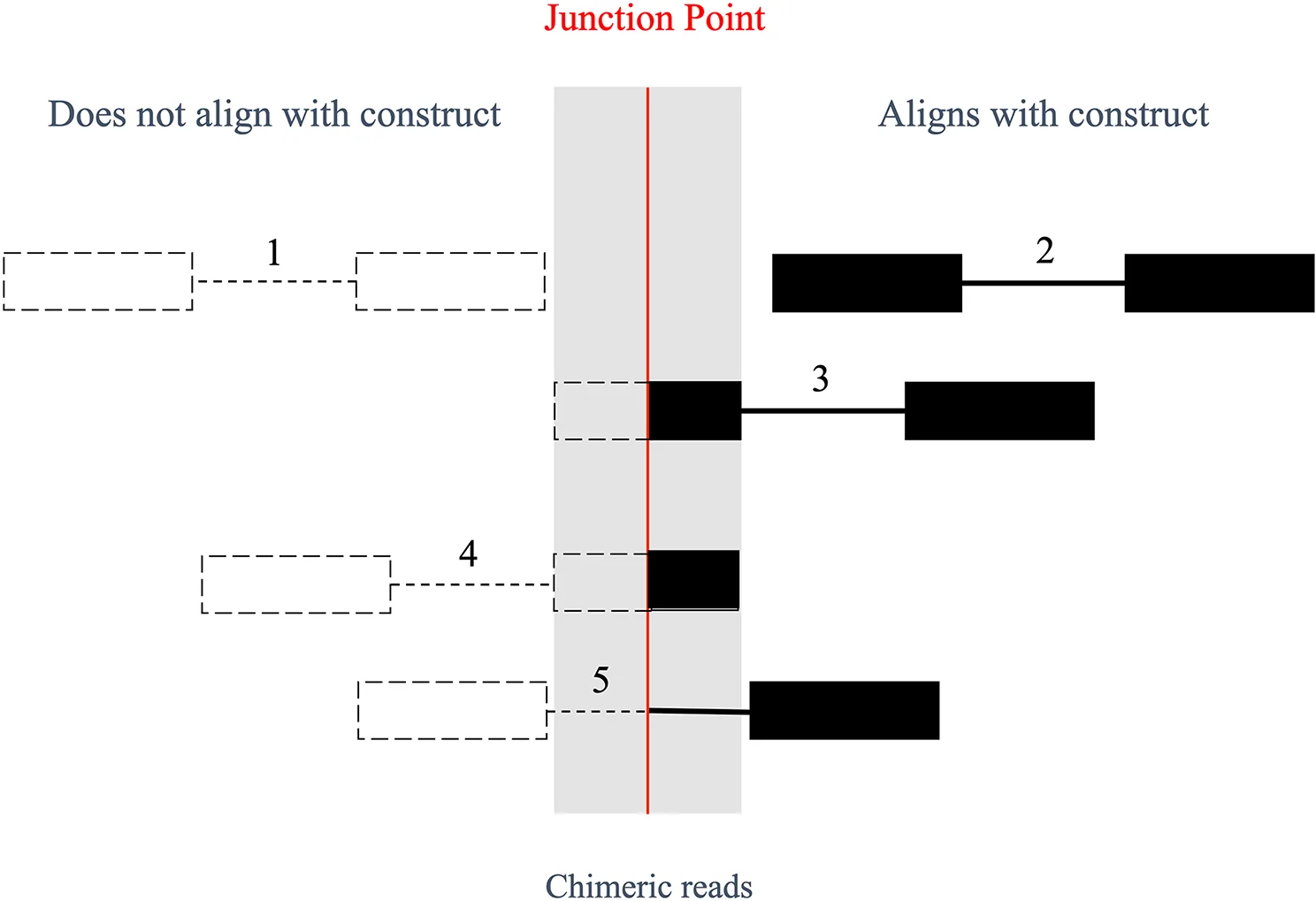
Illustration of the five categories of read alignments observed at construct-to-genome junction boundaries. When paired-end Illumina data from transgenic plants are mapped to the transformation construct near an insertion junction, read alignments fall into five categories based on their alignment to the construct. Read pairs are shown as connected boxes, with black boxes indicating construct alignment and dashed boxes indicating non-alignment. The schematic depicts the region near an insertion junction, with non-construct sequence to the left and construct sequence to the right. Categories are numbered 1–5, with categories 2–5 providing evidence of T-DNA presence and junction signatures as described in the text

Category 1 consists of non-aligning reads, i.e. reads originating entirely from the host genome, mitochondria, chloroplasts, or environmental DNA that do not align to the transformation construct. These constitute > 99.9% of total reads and are excluded from further analysis. Category 2 includes fully aligned paired reads, where both mates of a read pair fully align to the transformation construct. These reads confirm the potential presence of inserted DNA and are functionally equivalent to bands observed in Southern blotting.

The remaining three categories enable junction identification and copy-number determination. Category 3 comprises junction-spanning paired reads, where one mate fully aligns to the construct and the other partially aligns, with the partially aligned mate containing both construct and genomic sequence (chimeric read). These define junction boundaries. Category 4 consists of junction-spanning unpaired reads, i.e. single reads with partial alignment to the construct (chimeric reads) whose mate resides entirely in flanking genomic DNA. These accumulate at junction boundaries and provide critical evidence of construct-to-genome transitions. Category 5 includes full-length unpaired reads that fully align to the construct, but whose mates reside in flanking genomic DNA.

Together, Categories 2–5 provide comprehensive evidence of T-DNA presence (Category 2), precise junction boundaries (Categories 3–4), and insert structure (Categories 4–5). Understanding these read categories is essential for interpreting the mapping signatures demonstrated in the following examples.

### Control mappings and validation

Before interpreting specific transformation outcomes, systematic validation of control mappings should occur to ensure that any observations from the transformed sample represent genuine insertions rather than artifacts. Control mappings validate the accuracy and interpretability of WGS-based molecular characterizations. Data analysis begins by examining control samples for positive construct detection, presence or absence of endogenous cross-mapping, and single-copy locus (SCL) capture and depth. The transformation construct mapped to itself acts as a positive control for sequencability. Uniform coverage and consistent paired read alignment across the construct confirm that all regions are detectable. Apparent junction-like features (described below) at the ends of the linearized construct are artifacts caused by reads spanning the circular origin and do not represent true structural variation. These features, along with orientation shifts such as RL (right-left) reads, result from the artificial linearization and are expected (S1 Fig).

The non-transformed genome mapped to the transformation construct serves as a negative control (S2A Fig). This determines whether reads aligning to the construct in the transformed genome result from actual DNA insertion or from background homology. If reads from the conventional control map to the construct (S2B Fig), it indicates endogenous sequence similarity between the host genome and construct elements, which must be accounted for during interpretation. In practice, read pileups in the conventional control mapping can be used to discount comparable pileups in the transformed sample.

### Calculating achieved sequencing depth

Mapping each genome to a single-copy endogenous locus (SCL) provides an empirical measure of sequencing depth (S3 Fig) and is consistent with previously established methods [20]. Uniform coverage across this locus confirms that the expected depth has been achieved. Junction-like signals at the ends of the SCL reflect the boundaries of the reference sequence and do not represent insertions.

While the examples presented here use simulated data generated at a predetermined ~75× coverage, experimental datasets require empirical calculation of achieved sequencing depth. This calculation is performed by determining the average coverage across the SCL mapping, to serve as baseline depth for that dataset. Command-line tools such as SAMtools can be applied directly to the mapped SCL data to provide straightforward calculation of per-base coverage [25]. Alternatively, visualization tools may allow direct export of summary statistics or per-nucleotide coverage data that can be processed to the same end [31, 32]. The empirically determined SCL depth then serves as the reference for all coverage-based interpretations.

### Junction identification and characterization

After observing paired reads mapping to T-DNA regions (confirming the presence of inserted DNA), the next step is to identify precise junction boundaries using chimeric reads. In IGV, chimeric reads are visually recognized by their characteristic pattern of multicolored soft-clipped bases extending beyond the construct-aligned portion of the read. These soft-clipped regions represent the portions of chimeric reads that extend beyond the construct boundary into adjacent genomic flanking sequence. At true junction sites, these reads accumulate into localized pileups of unpaired and paired chimeric reads all terminating at the same genomic position.

Junction pileups are identified by three key visual features: (1) a sharp transition in coverage depth marking the insert boundary, (2) accumulation of chimeric reads with soft-clipped bases (unaligned portions displayed as multicolored bases in IGV) at the boundary position, and (3) a pileup of unpaired reads mapping just inside the construct and up to the termination point.

True junctions typically display ≥ 50% of achieved sequencing depth as localized, high-density pileups of unpaired and chimeric reads terminating at a single coordinate, combined with sustained paired-read coverage. Localized coverage reduction at junction boundaries is expected and normal, as reads lose mate-pair context and progressively shorter chimeric reads fail to meet minimum mapping thresholds.

Once junction locations are visually confirmed in the genome browser, the chimeric reads can be extracted and aligned using tools like Clustal [33] or other similar multiple sequence alignment programs. The soft-clipped portions of these reads, which terminate at a consistent coordinate, demarcate the precise construct-to-genome transition point (S11 Data, S12 Data). Consensus sequences extracted from these soft-clipped regions define the genomic flanking sequence, which can then be mapped to a reference genome using any common pairwise aligner such as FASTA [28] or BLAST [34] to determine chromosomal insertion coordinates (S13 Data). For events with multiple insertions at similar construct coordinates, alignment of chimeric reads from each pileup reveals distinct flanking sequences that enable disambiguation of which junctions belong together (S12 Data).

Junction reads typically appear at the terminal ends of inserted DNA. However, if junction-like features occur within the insert region or construct backbone, they may indicate structural rearrangements, co-insertions of additional DNA fragments, endogenously sourced reads with sequence homology to the construct, or other anomalies requiring careful interpretation. When internal junction signatures cannot be accounted for by control mappings (e.g., endogenous homology to the construct), the chimeric reads should be extracted and subjected to sequence-level analysis using alignment tools to determine the nature of the structural variation.

Examining the sequence composition of soft-clipped portions of chimeric reads provides critical diagnostic value for this analysis. At true construct-to-genome boundaries, soft-clipped sequences derive from genomic flanking DNA and align to the host genome reference. In contrast, at duplicated construct elements displaying cross-mapping artifacts, any chimeric reads present contain soft-clipped sequences matching other construct regions rather than genomic DNA. This distinction helps confirm whether observed chimeric signals represent genuine insertion boundaries or mapping artifacts from sequence homology within the construct.

### Coverage and paired-read features for structural interpretation

Junction capture and sequence analysis alone are sufficient for successful molecular characterization, and coverage analysis serves as helpful validation rather than a requirement. When sequencing is reasonably uniform across the genome, T-DNA coverage depth can be interpreted proportionally relative to the empirically determined SCL depth as a corroborating line of evidence. The proportional relationship between T-DNA depth and SCL depth is most straightforward in homozygous inbred diploid organisms, as established for the simulated examples in this manuscript. In other contexts, including hemizygous events, hybrid backgrounds, and polyploid species, the relationship between observed depth and copy number is influenced by ploidy, zygosity, and genome background; therefore, depth ratios should be treated as approximate supporting evidence that results are in line with expectations rather than standalone copy-number determinations. For this reason, depth observations are most informative when considered alongside junction signatures, mate-pair behavior, and coverage uniformity, with junction analysis providing the definitive structural interpretation. In samples with highly variable amplification, non-uniform library preparation, or biased genomic representation, coverage-based estimates become unreliable and should be interpreted with considerable caution, if utilized at all.

Coverage can also be evaluated for uniformity and relative proportions across three scales: (1) element (individual promoters, terminators, or coding sequences), (2) regional (contiguous spans within the T-DNA), and (3) whole-insert (entire T-DNA or construct). Comparing coverage proportionality and patterns across these scales, alongside junction positions, aids in disambiguating complex insertion events.

Paired-read mapping characteristics provide additional structural insights. Standard Left-Right (LR) orientation, where mate pairs point inward toward each other, reflects expected mapping. Anomalous orientations such Right-Left (RL), Left-Left (LL), or Right-Right (RR) may indicate structural rearrangements, duplications, or arise as cross-mapping artifacts from duplicated construct elements. In size-selected libraries, consistent mate-pair spacing within the expected range (e.g., 400–550 bp) supports intact integration, while deviations may indicate potential rearrangements or duplications (see Insertions with Unintended Structural Arrangements section and S7 Fig).

By systematically evaluating junction signatures, coverage patterns, and paired-read features, analysts can efficiently interpret WGS-based molecular characterization data.

### Interpretive examples

Representative examples demonstrate how WGS signatures manifest across diverse transformation outcomes, progressing from ideal single-copy insertions through increasingly complex scenarios. Each example illustrates how the interpretive principles outlined above can be applied in practice. Depth observations in the examples below assume the homozygous inbred diploid background described in Methods; see Coverage and Paired-Read Features for interpretation in other contexts.

### Single-copy T-DNA insert

The goal of a typical transformation is to achieve a single copy of the intended T-DNA inserted at a single locus, without transfer of construct backbone and with minimal genomic disruption. The WGS signature of such an event is characterized by uniform coverage across the T-DNA region, consistent paired reads, and two distinct junctions as defined by chimeric reads at terminal ends of the T-DNA.

Clear termination points at both T-DNA ends confirm precise junctions (Fig. 3). The absence of reads mapping to the construct backbone confirms that no backbone sequences were inserted. Coverage depth across the T-DNA is consistent with the SCL, corroborating the single-copy insert determination inferred by the junction signatures (S3 Fig).

**Fig. 3:**
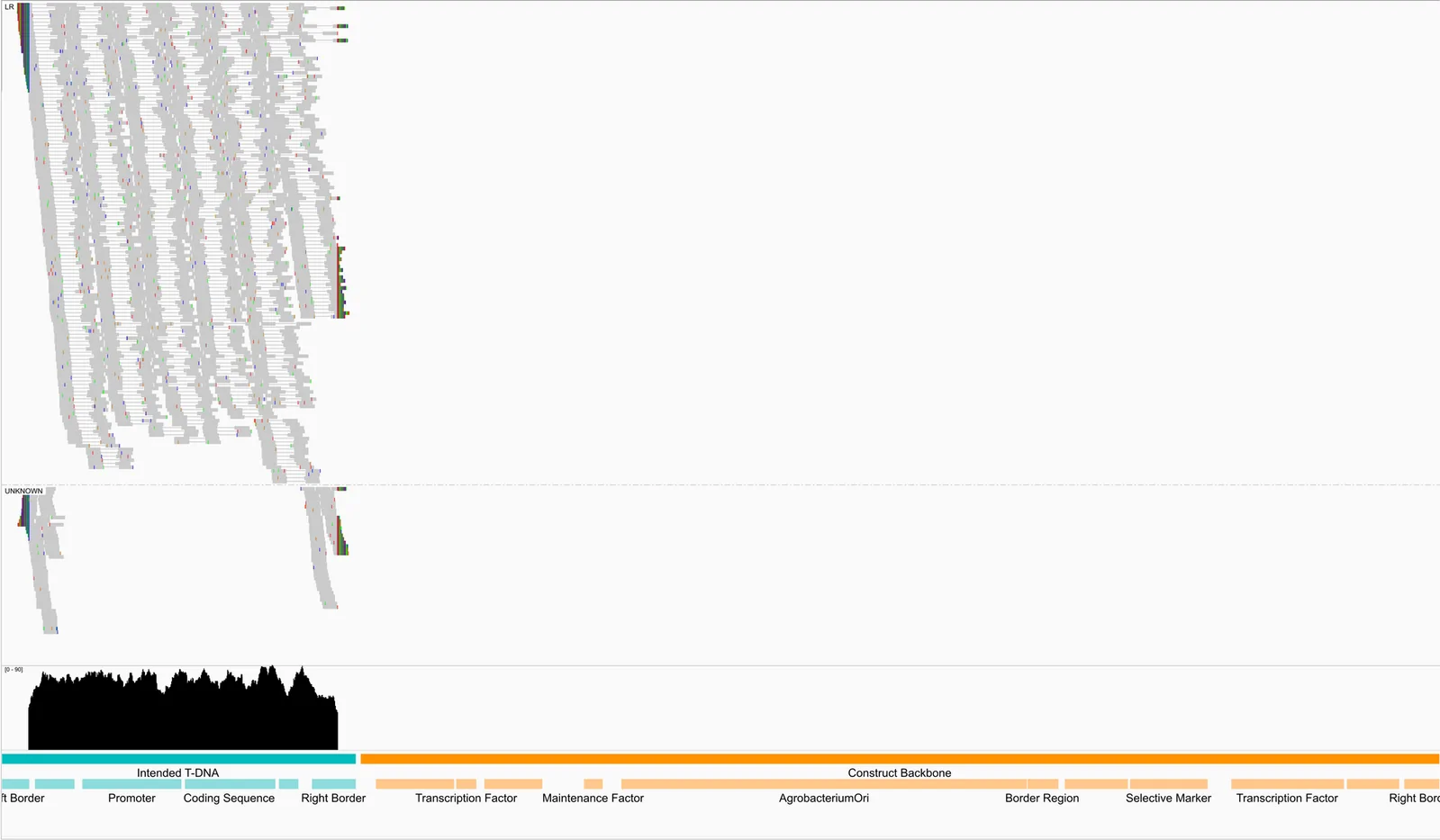
Mapping of a single-copy T-DNA insert. Paired reads span the intended T-DNA region, confirming its presence. Unpaired and chimeric reads accumulate at both ends, indicating junctions. Coverage across the insert is uniform, and consistent with the SCL coverage depth, averaging ~75×, with no reads mapping to the construct backbone

Once junctions and T-DNA integrity are visually confirmed, chimeric reads which span into the adjacent genomic flank can be extracted and aligned using tools like Clustal to determine the exact junction sequences and flanking genomic DNA (S11 Data) [20, 33]. 

### Event with multiple insertions

 The WGS signature of multiple T-DNA insertions resembles that of a single insert but includes multiple junctions and increased coverage depth. In the example provided (Fig. 4), paired reads span the intended T-DNA, while unpaired and chimeric reads accumulate at both ends with two abrupt termination points at each border, indicating two distinct junctions. No reads map to the construct backbone, confirming its absence.

**Fig. 4:**
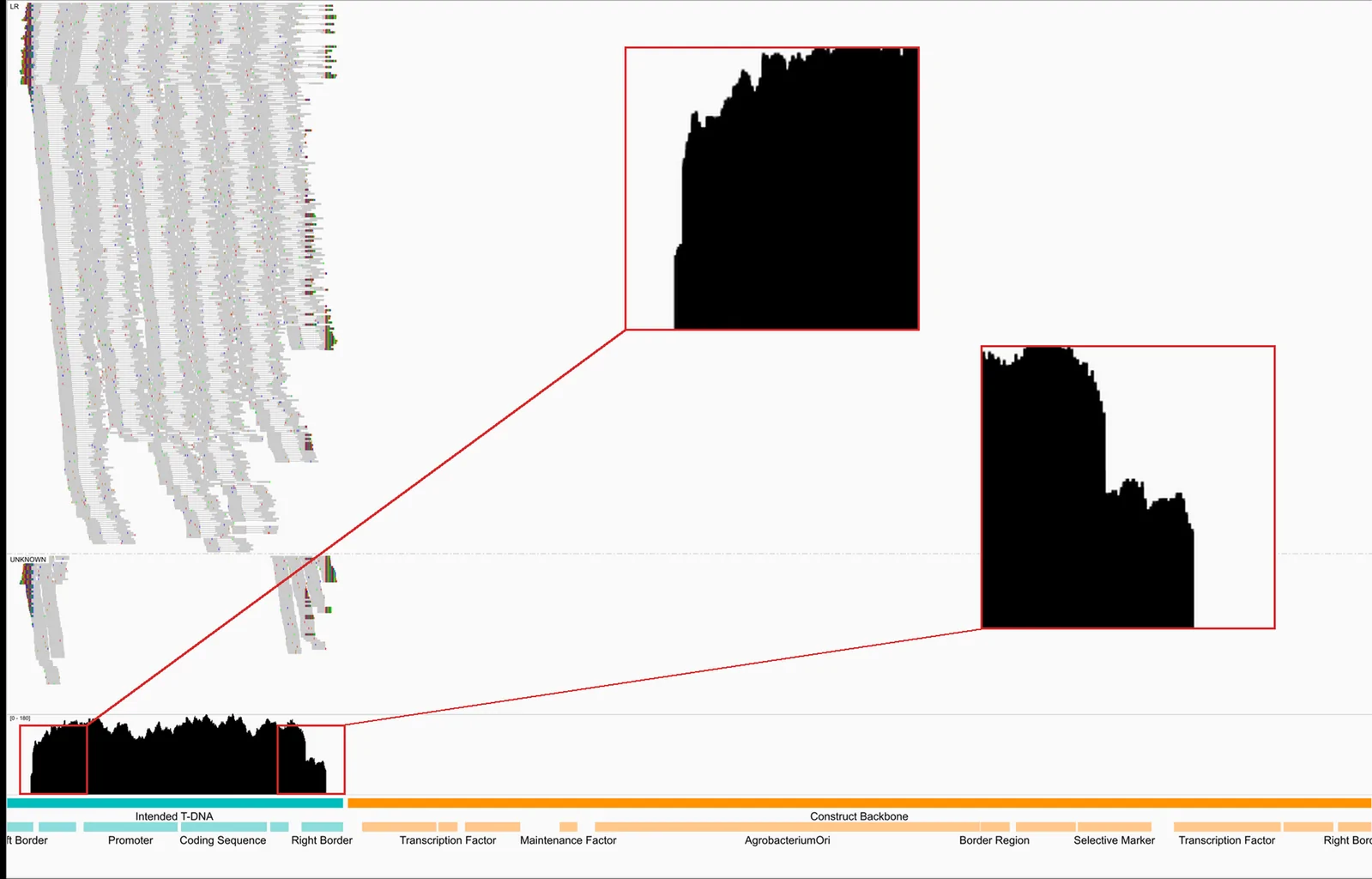
Mapping of a multiple (double) insert event. Paired reads span the intended T-DNA region. Unpaired and chimeric reads accumulate at both ends, indicating four distinct junctions (two per border). The left border junctions are narrowly offset, while the right border junctions exhibit a broader junction offset resulting in a step-like appearance in the depth, consistent with two independent single-copy insertions contributing additively to coverage depth. No reads map to the construct backbone

Coverage depth across the T-DNA is approximately 150×, broadly consistent with the SCL depth scaled for two insertions under the homozygous inbred diploid conditions established for these simulations, corroborating the junction-based interpretation of two independent single-copy insertions.

Careful analysis is required when junctions are narrowly offset (e.g., left junction Fig. 4), as the graphical rendering may obscure multiple junction sites. To determine junction number, reads terminating at similar positions should be aligned to verify whether they share identical flanking genomic sequences (indicating a single junction) or display distinct flanking sequences (indicating multiple junctions). In the Fig 4 example, four junctions are identified with this strategy: two at the left border and two at the right border, representing two complete T-DNA insertions.

While junction locations can be visually identified in the genome browser, flanking sequence analysis is required to determine chromosomal insertion sites and pair junctions from multiple independent insertions. This involves: (1) Extracting chimeric reads from each junction cluster, (2) Aligning to identify distinct flanking sequences (S11 Data, S12 Data), (3) Mapping each consensus flanking sequence to the reference genome to determine chromosomal coordinates, and (4) Pairing junctions based on chromosomal co-localization (S13 Data). This enables unambiguous reconstruction of each independent insertion event.

### Homologous element shared between T-DNA and backbone

Certain genetic elements may be intentionally present both within the intended T-DNA and in the non-transferred plasmid backbone, resulting in sequence homology between these two regions of the reference construct. This homology can produce characteristic mapping artifacts that must be distinguished from true backbone integration. In the example shown (Fig. 5), the intended T-DNA is inserted as a single copy, with junction pileups confirming its boundaries. Paired reads originating from this single inserted copy also align to the homologous region in the backbone due to sequence identity, generating a pyramidal coverage profile.

**Fig. 5:**
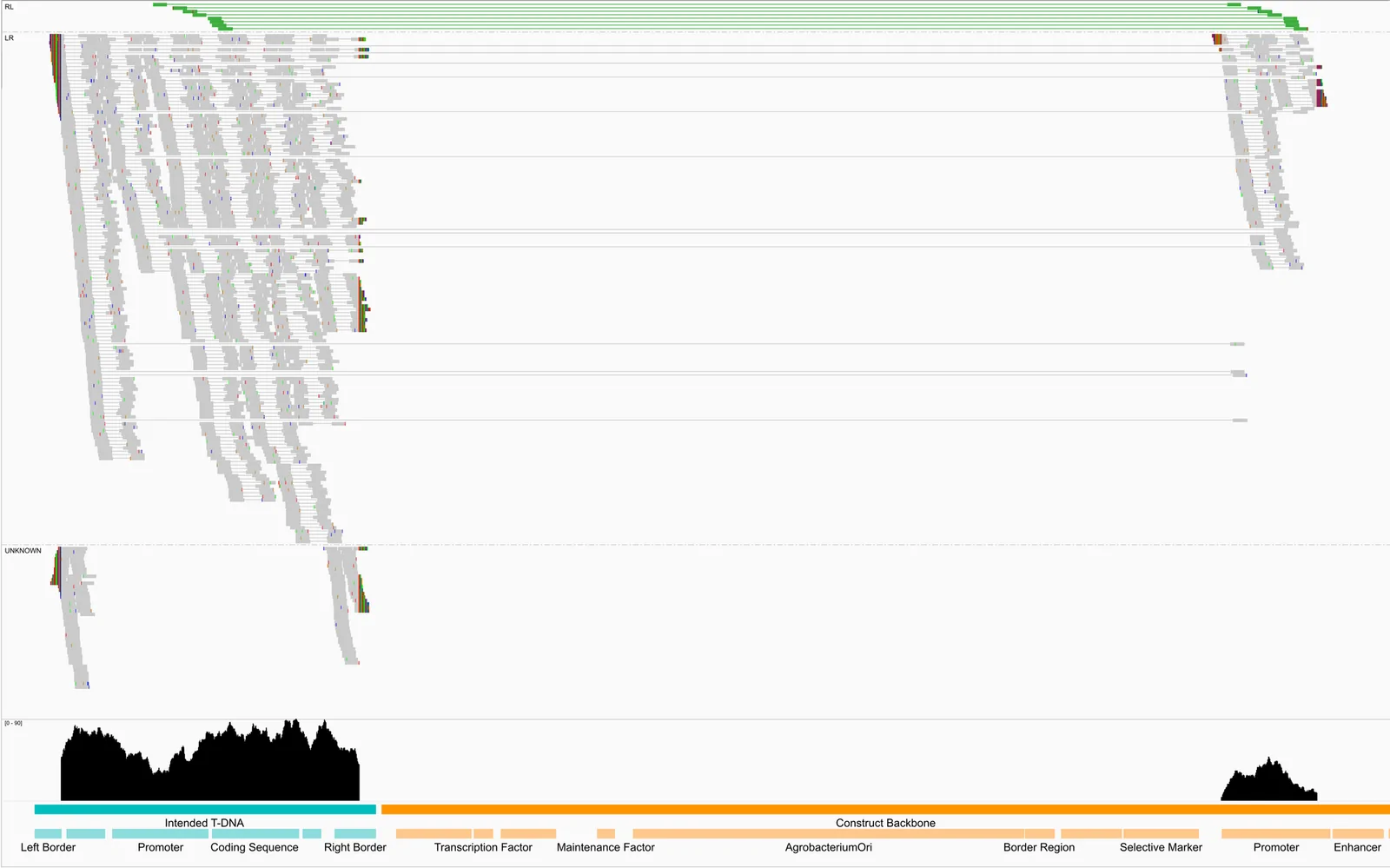
Single-copy T-DNA with ambiguous read distribution across duplicated backbone promoter element. Single-copy T-DNA insertion with expected junction pileups at the borders and complete coverage across the inserted region. Reads from the inserted promoter also map to its homologous copy in the non-transferred backbone, producing a characteristic pyramidal coverage profile and an apparent relative dip in the T-DNA promoter region. Soft-clipped sequences in the backbone region match adjacent T-DNA elements rather than genomic flanking DNA, confirming these arise from cross-mapping of reads originating at the true T-DNA insertion. The absence of chimeric reads at the duplicated element with genomic flanking sequences confirms no genuine construct-to-genome junction exists at the backbone

This pyramidal shape arises because reads fully contained within the homologous segment lack unique flanking context and map with equal likelihood to either location, whereas reads extending into adjacent unique sequence are preferentially anchored to the T-DNA copy. This results in an approximately 50/50 distribution across the two positions, expected from random alignment selection when reads can map equally well to multiple locations (see Limitations and Best Practices section for aligner behavior details). Additionally, reads mapping between the T-DNA and backbone copies display characteristic anomalous features: Right-Left (RL) orientation (rather than the standard inward-pointing Left-Right configuration), combined with extended insert sizes visible as long connecting lines in IGV. Soft-clipped sequences in reads mapping to the backbone promoter match adjacent T-DNA sequence rather than genomic flanking DNA, further confirming their origin from the true T-DNA insertion. This diagnostic feature of soft-clipped sequences matching construct elements rather than genomic DNA is the definitive evidence distinguishing cross-mapping artifacts from genuine multi-locus insertions. Similar cross-mapping can occur when identical elements are duplicated by design at multiple positions within the T-DNA itself, producing comparable artifacts but with signatures entirely contained within T-DNA boundaries rather than spanning to the backbone region. The apparent ~50% coverage drop over the homologous element is an artifact of read distribution, as the single inserted region is being mapped to two locations. When accounting for reads split between both copies, overall T-DNA coverage remains consistent at ~75×. Collectively, these features confirm cross-mapping between duplicated regions rather than indicating a true secondary insertion.

### Events containing endogenous sequences

Transformation constructs may include elements with homology to endogenous sequences, which can produce misleading WGS signatures. These signatures often resemble small secondary insertions within the T-DNA due to elevated coverage over the homologous region. In the example shown (Fig. 6), a full intact single T-DNA signature is present. However, additional junction-like features appear around the promoter element in the T-DNA, accompanied by increased coverage (~150×) and chimeric/unpaired reads, indicative of a potential insertion.

**Fig. 6:**
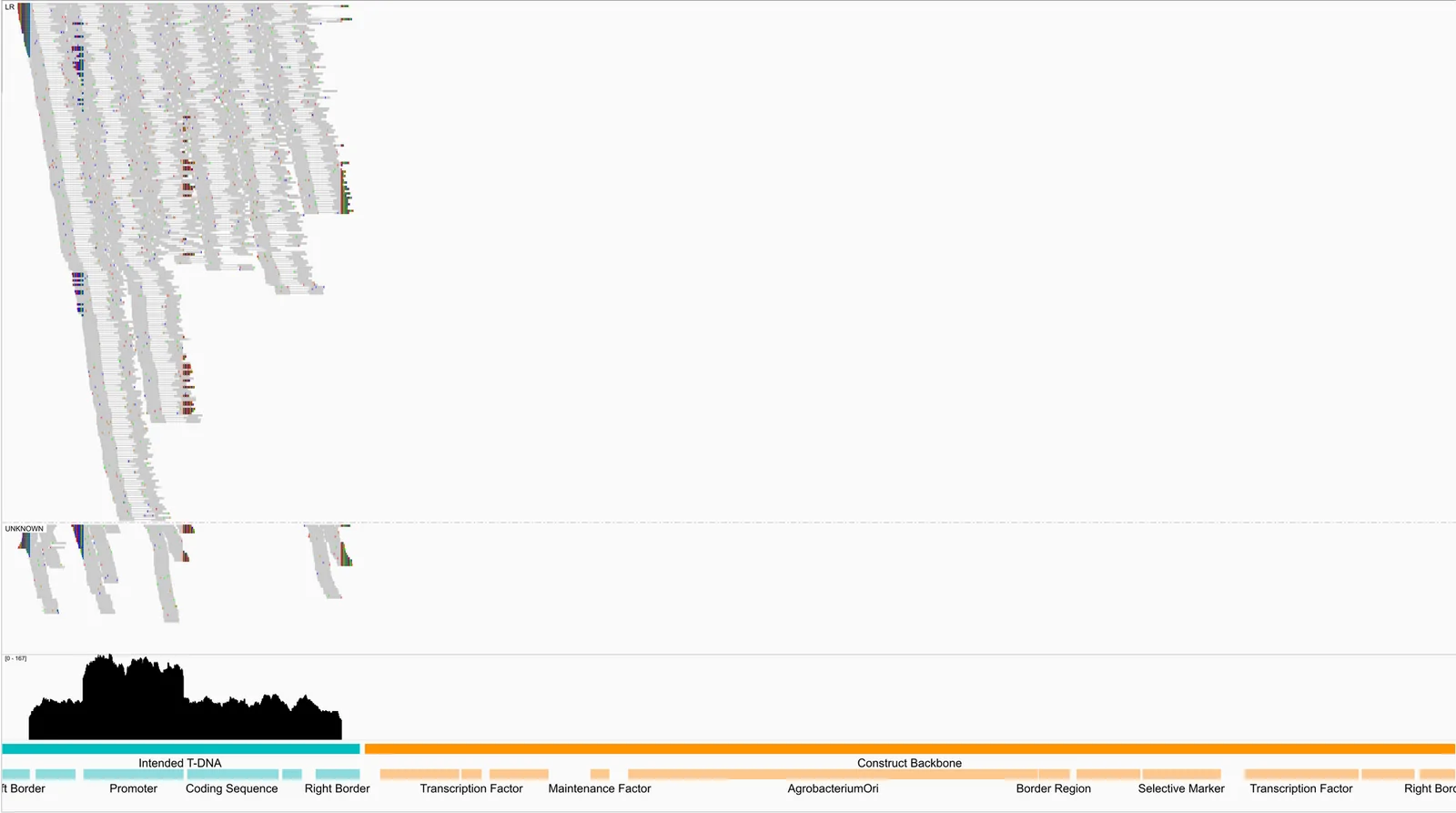
T-DNA insert with elevated promoter coverage mimicking secondary insertion signature. Paired reads span the T-DNA, with chimeric reads strongly confirming terminal junctions. Elevated coverage (~150×, representing ~2-fold over background) and junction-like features around the promoter element in the T-DNA at a high level display characteristics of a secondary insertion. However, this signature is also present in the conventional control (Fig. 7), confirming the promoter is an endogenous sequence. Coverage across the rest of the T-DNA remains uniform at ~75×

To determine whether this elevated promoter signal represents a genuine secondary insertion or endogenous homology, the negative control mapping is essential. Mapping reads from the control genome to the transformation construct reveals the same promoter-associated signature (Fig. 7), confirming that the promoter sequence is endogenous and not a second insertion. The junction-like signals at the ends of the endogenous region simply reflect the boundaries of the reference. This highlights the importance of including a conventional control to identify host native sequences that may map to the construct and prevent misinterpretation of WGS data.

**Fig. 7:**
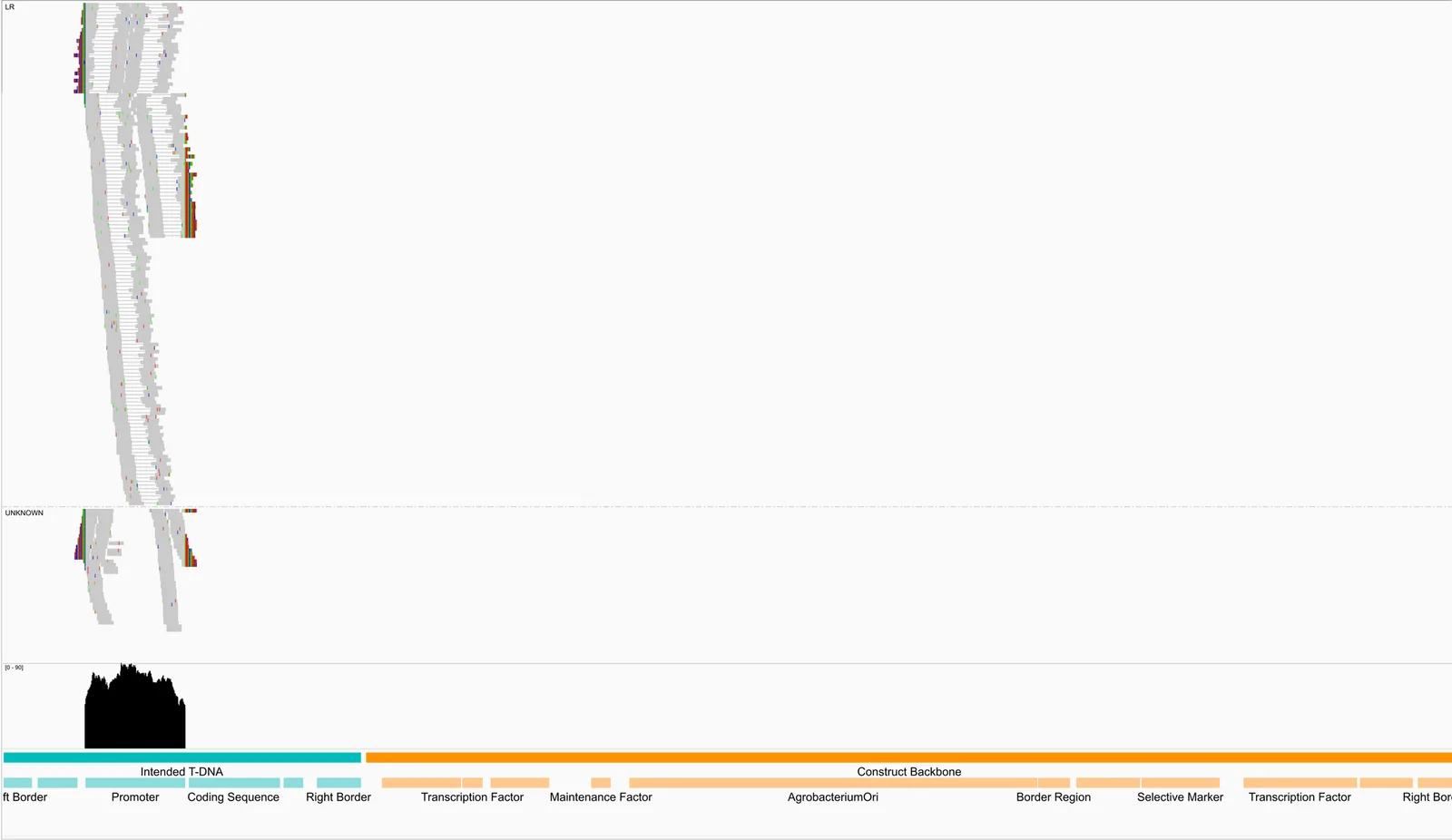
Negative control verifying promoter sequence is endogenous to the host genome. Mapping the conventional control to the transformation construct shows paired reads aligning to the promoter region (same as Fig. 6), producing a junction-like signature and distinct coverage (~75×, matching genome-wide depth) despite no actual insertion. This confirms the promoter is an endogenous sequence, explaining the elevated coverage observed in the transformed event

Any construct region showing elevated coverage and junction-like features in both the transformed sample and the conventional control should be attributed to endogenous homology rather than insertion, regardless of signal strength. This example demonstrates the critical importance of the negative control mapping strategy established in Table 1 and discussed in the Control Mappings section.

### Insertions with unintended structural arrangements and polymorphisms

While the goal of transformation is a clean, single-copy T-DNA insertion, unintended outcomes occur with measurable frequency and exhibit characteristic WGS signatures that enable their detection and structural resolution.

Backbone Co-integrations: Unintended backbone sequences may integrate alongside the T-DNA, either contiguously at a single locus (S4 Fig) or at independent genomic locations (S5 Fig). These events are identified by junction pileups occurring outside the intended T-DNA borders, combined with single-copy-level coverage across both the T-DNA and backbone regions. The presence of clearly defined junctions flanking the backbone segment (S5 Fig) distinguishes true co-integration from cross-mapping artifacts.

Sequence Polymorphisms: Sequence variations relative to the transformation construct (including single nucleotide polymorphisms (SNPs), insertions, and/or deletions) manifest as consistent mismatches, gaps, or colored bases in aligned reads (S6 Fig). These polymorphisms are validated by extracting consensus sequences from the mapped reads and performing pairwise alignment against the reference construct. In IGV, SNPs appear as consistent alternate base colors across multiple reads, insertions as purple “I” markers, and deletions as black lines with coverage dips.

Tandem Fragment Co-insertions: Tandem fragments, where a segment of T-DNA is physically duplicated and co-inserted at a junction boundary (S7 Fig), produce characteristic signatures including localized elevated coverage over the duplicated span, anomalous mate-pair orientations (RL, LL, RR rather than LR), and condensed or extended insert sizes beyond the expected range. Unlike cross-mapping artifacts from intentionally duplicated construct elements, these tandem co-insertions show localized elevated coverage over the duplicated region.

Familiarity with these signatures is essential for comprehensive molecular characterization, as off-type events, while undesirable for commercial development, provide valuable information about transformation mechanisms and must be accurately characterized during screening and event selection.

### Integrated example of multiple signatures in a single dataset

Real-world WGS characterization datasets may contain combinations of expected structural elements, duplicated construct regions, low-level contamination, and other features, all of which must be correctly interpreted within a single analysis. The following example (Fig. 8) demonstrates how the interpretive framework presented here enables systematic dissection of complex datasets containing multiple concurrent signatures. Successful interpretation requires systematically evaluating each region of the construct mapping while considering junction quality, coverage patterns, mate-pair behaviors, and control mappings in light of the observation types discussed throughout this work.

**Fig. 8:**
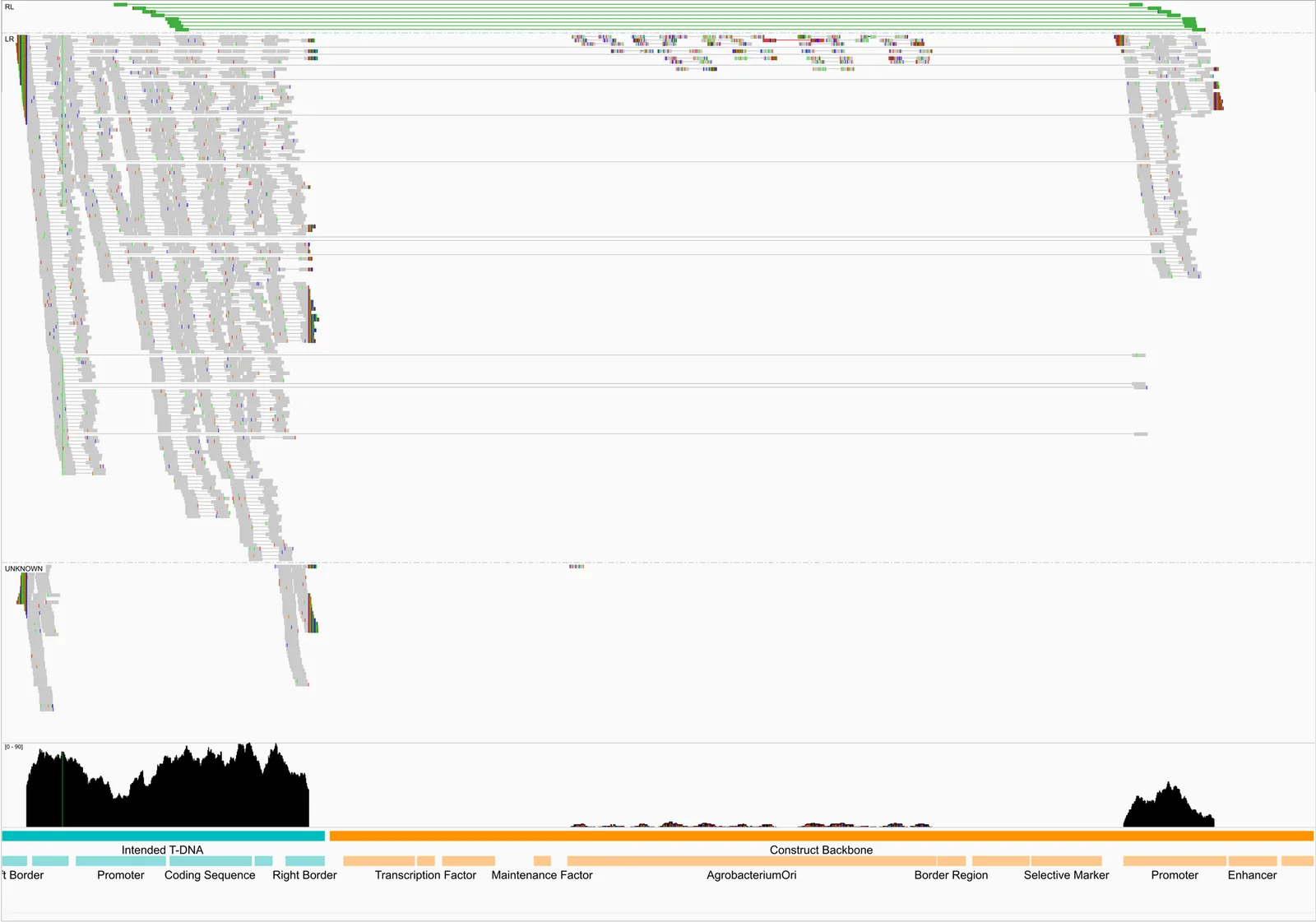
Integrated example with insertion, sequence variation, duplicated element, and bacterial contamination. Real-world WGS characterizations often present multiple concurrent signatures requiring systematic interpretation. This dataset demonstrates such complexity: junction-spanning reads at both borders confirm a single-copy T-DNA insert. A single nucleotide polymorphism is visible in the mapped reads. The duplicated promoter in the backbone creates a pyramidal coverage pattern from ambiguous read distribution. Low-depth reads (~3×) map sporadically to the Agrobacterium ori element with sequence mismatches, indicating minor bacterial contamination. Overall T-DNA coverage remains consistent at ~75× when accounting for split mappings

In the example shown (Fig. 8), the intended T-DNA is present with junctions consistent with a single-copy insert. Junction-spanning reads at both borders display the characteristic high-density pileups with progressive truncated soft-clipping patterns, confirming authentic insertion boundaries. A single nucleotide polymorphism relative to the construct reference sequence is observed in the mapped reads (visible as consistent colored bases across multiple reads), representing an unexpected sequence variation.

Coverage across the T-DNA is mostly uniform (~75×), except for a dip over the promoter element. This dip corresponds to a duplicated promoter in the backbone, which recruits reads away from the T-DNA copy due to sequence homology, resulting in the pyramidal coverage pattern discussed previously (Fig. 5). The cross-mapping arising from the duplicated element is further confirmed by both properly paired and discordant (RL) long inner-mate distance reads linking the homologous regions. When accounting for reads split between both copies, overall T-DNA coverage remains consistent at ~75×.

Additional reads map to the *Agrobacterium* ori element in the backbone. Unlike true junctions, these reads display hallmarks of contamination: sporadic alignment patterns, soft-clipped reads with inconsistent termination points, and sequence mismatches visible in IGV as colored bases, purple “I” insertion markers, and black bars indicating deletions throughout mapped reads. Coverage depth is minimal (~3×, representing only 4% of experimental depth) and lacks the concentrated chimeric pileups characteristic of genuine junctions. The relative depth of contaminant sequencing depends on contamination levels during library preparation. When contamination approaches or exceeds experimental depth, or confounds interpretation in any way, DNA extraction and sequencing should be repeated with enhanced sterility protocols (additional contamination examples in S8 Fig, S9 Fig).

Overall, this dataset demonstrates how WGS can resolve complex transgenic structures, even in the presence of contamination and duplicated elements. The systematic evaluation of junction quality, coverage proportionality, mate-pair characteristics, and cross-referencing with the duplicated element pattern enables confident characterization: a single-copy T-DNA insert with a minor SNP, correctly accounting for cross-mapping artifacts from the duplicated promoter and distinguishing the true insertion from low-level bacterial contamination.

### Consistency of simulated and real-world mapping signatures

The simulated examples presented in this study were designed to illustrate characteristic read‑mapping signatures associated with different T‑DNA insertion outcomes under controlled conditions. By isolating individual features such as junction pileups, coverage transitions, mate‑pair behavior, and backbone signals, these examples provide a clear and reproducible foundation for interpreting mapped‑read patterns.

To demonstrate that the signatures illustrated using simulated data are consistent with those observed in real sequencing data, we examined a whole‑genome Illumina paired‑end dataset corresponding to a single‑copy T‑DNA insertion event in Roundup Ready 2 Yield soybean (MON 89788). Reads were mapped to the original transformation construct used for event generation (Fig. 9). The region of the construct to which reads aligned corresponds exactly to the T‑DNA sequence reported in the publicly available insert record deposited under GenBank accession HW805122.1. The accompanying control mappings for this dataset, including the conventional parent line mapped to the transformation construct (S10A Fig) and the single-copy locus mapped for empirical depth calibration (S10B Fig), demonstrate that the full control framework established in this manuscript performs consistently with real sequencing data.

**Fig. 9:**
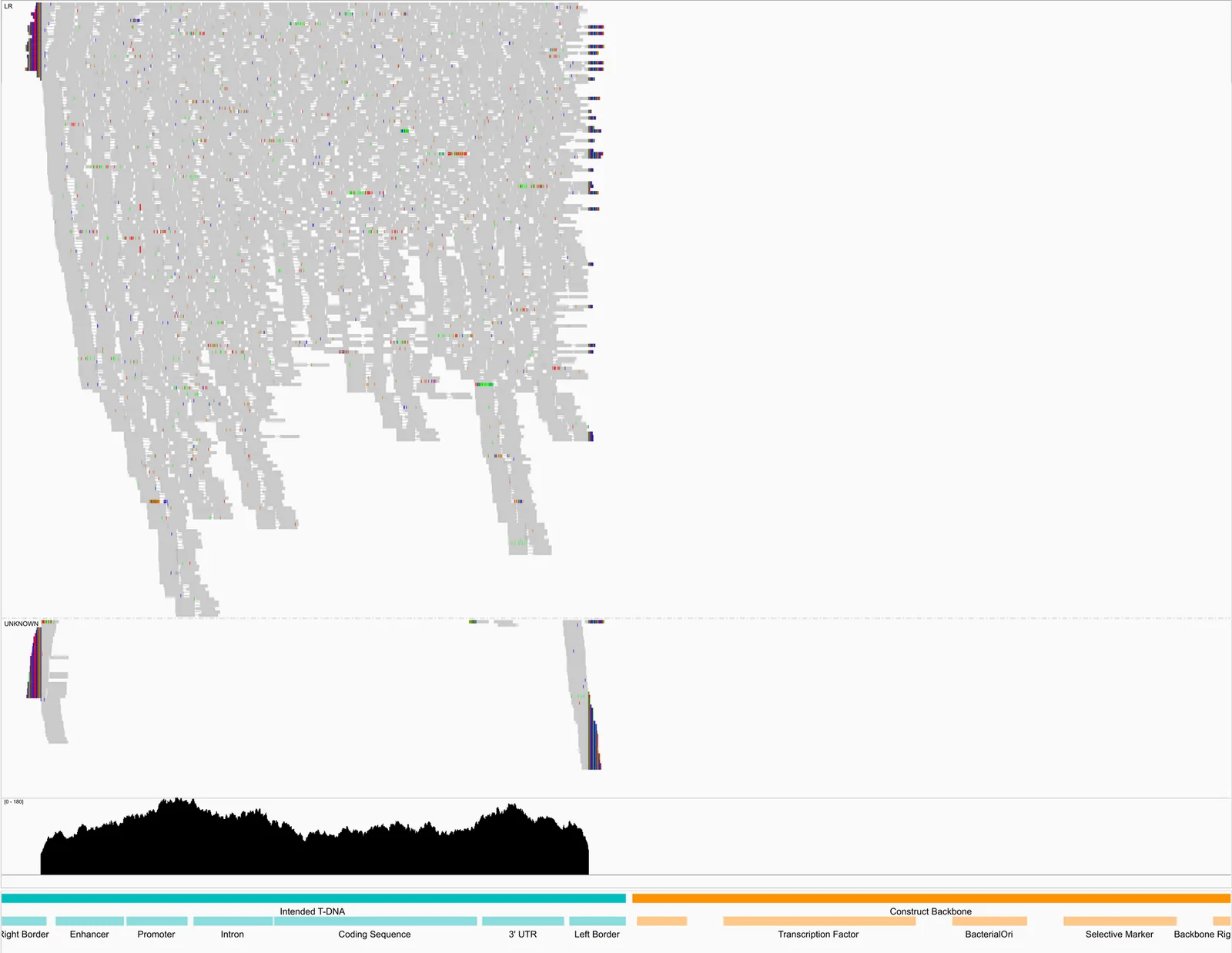
Real‑world example of a single‑copy T‑DNA insertion showing characteristic mapped‑read signatures. Whole‑genome Illumina paired‑end reads from Roundup Ready 2 Yield soybean (MON 89788) mapped to the original transformation construct. The aligned region corresponds to the T‑DNA portion of the publicly available insert sequence (GenBank accession HW805122.1). Insert shows left and right T‑DNA borders with abrupt coverage termination and localized pileups of junction‑spanning chimeric reads with soft‑clipped bases extending into flanking genomic sequence. No read coverage is observed over backbone‑only regions of the construct

The observed mapping patterns mirrored those shown in the simulated single‑copy examples. Coverage across the T‑DNA region was uniform relative to the endogenous single‑copy locus control, and two discrete junction boundaries were evident at the left and right borders. Each boundary was marked by sharp coverage termination and localized pileups of junction‑spanning chimeric reads with soft‑clipped bases extending into flanking genomic sequence. No reads mapped to backbone‑only regions of the construct, and no additional junction signatures were detected, consistent with a single intact insertion event.

Simulated datasets might be perceived as overly idealized relative to practical sequencing data. However, when appropriate controls and standard sequencing and mapping procedures are applied, the fundamental read‑mapping signatures observed in real whole‑genome sequencing data can be nearly indistinguishable from those produced under simulation. This is particularly true for regulatory‑grade whole‑genome sequencing datasets generated under validated laboratory and bioinformatic workflows. This comparison illustrates that the signatures defined using simulated examples reflect genuine alignment behavior, and that simulated data provide an effective and transparent foundation for diagnostic interpretation that transfers directly to real sequencing datasets.

## Limitations and best practices

### DNA extraction and library preparation

Environmental contamination during DNA extraction or library preparation can produce artifactual backbone mapping. When sequencing at high depth, sporadic low-level reads may appear across construct regions even in the absence of true insertions, resulting from sequencing errors, partial endogenous homology, low-complexity regions, environmental contamination, index hopping during multiplexed runs, or any combination thereof [12, 35–41]. Truly artifactual mappings, i.e. those arising from sequencing errors, repetitive elements, and alignment ambiguities, rarely exceed ~5% of experimental depth, whereas contamination levels scale with contaminant concentration and may approach or exceed experimental depth. The critical distinction is recognizing that these sporadic read mappings lack the concentrated chimeric pileups characteristic of genuine junctions, as well as contiguous coverage over their lengths. Using tissue amenable to rigorous surface sterilization and applying effective decontamination protocols, including ensuring sterile lab environments when handling the chosen starting material, greatly reduces environmental contamination risk.

Size-selecting sequencing libraries helps maintain consistent mate-pair spacing, making deviations easier to detect. With a size selected library, abnormal mate-pair spacing readily indicates potential structural rearrangements or identifies duplicated elements (S7 Fig).

### Sequencing and read processing

Recommended sequencing depth depends on the intended application. For research or high-throughput screening purposes, successful molecular characterization is achievable at depths as low as 10–20× [14, 17, 19]. For regulatory submissions EFSA’s Technical Notes recommend a minimum read depth of 40× for determination of the insert sequence and genomic flanking regions [9, 10]. Because junction sites exhibit localized coverage reductions due to mate-pair loss and chimeric read mapping thresholds, 75× genome-wide coverage might be considered the practical minimum for reliably meeting this regulatory threshold in a single workflow. This depth is consistent with previous work [20], which justified 75× as sufficient for both characterization and to ensure complete capture of the host genome. Higher depth (≥100×) provides additional confidence for complex insertions or closely spaced junctions and increases the likelihood of meeting the 40× recommendation across all junction sites.

Illumina sequencing itself can introduce confounding variables such as non-uniform amplification, positional losses of quality, missed nucleotide calls, sequence-specific errors, GC bias, PCR and optical duplicates, and chimeric reads generated during library preparation or amplification [35, 36]. Many genomic workflows address these through quality trimming, which benefits applications like *de novo* assembly and variant detection [42]. However, for WGS molecular characterization, trimming can hinder junction detection by removing informative chimeric reads. Since low-quality reads typically fail to align anyway, trimming is unnecessary and may reduce junction sensitivity. Consequently, retaining untrimmed reads and sequencing at high depth is preferred, increasing likelihood of capturing junction-spanning reads and improving insert boundary resolution even when some reads contain spurious mismatches.

### Mapping and visualization

While no transgene-specific analysis software currently exists, the broad suite of general tools developed for WGS data processing provides ample resources for molecular characterization. Any commonly used short-read aligner (e.g., Bowtie2, BBMap, BWA, minimap2) [22, 26, 43, 44], can be sufficiently tailored to align WGS data for molecular characterization. IGV is a robust visualizer which has successfully been used for molecular characterization [15, 18, 19], yet other visualizers are equally sufficient for such an analysis (e.g Tablet, UGENE) [27, 31]. Default settings and visualization behaviors can vary across software, and it is imperative that investigators understand how these differences may influence their observations relative to the examples shown here. Regardless of software choices, ensuring data quality and interpretive rigor remains essential for reliable conclusions.

When transformation constructs or T-DNA regions contain multiple redundant sequences, aligner behavior with respect to multi-mapping reads becomes an important interpretive consideration. Short-read aligners vary in how they handle reads with multiple equally good placements, including whether additional placements are reported as secondary alignments, how chimeric mappings are represented, and how mapping quality scores reflect alignment ambiguity. When identical or highly similar sequence elements are present within a transformation construct, reads may be distributed across multiple positions rather than concentrated at a single locus. The pyramidal coverage pattern shown in Fig. 5 is a characteristic example, arising when reads are apportioned between duplicated elements. In such cases, localized coverage reductions should be interpreted in the context of multi-mapping rather than as evidence of additional insertions, with mapping quality, mate-pair behavior, and junction signatures providing supporting context. A similar consideration applies in polyploid species, where homologous chromosome sets can produce ambiguous mapping at the genome scale; in such backgrounds, junction analysis remains the most reliable basis for structural characterization, with coverage uniformity and copy-number inference more strongly affected.

An alternative strategy involves constructing a joint reference by combining the transformation construct and host genome into a single mapping target, allowing the aligner to resolve construct-versus-endogenous ambiguity in a single pass. Split-aware aligners such as minimap2 [44] generate supplementary alignments by default, making chimeric junction reads immediately discernable in visualization tools without a separate re-alignment step. The diagnostic signatures described throughout this manuscript manifest equivalently whether reported as soft-clipped reads or supplementary alignments, and the interpretive framework applies regardless of alignment strategy. However, joint-reference mapping increases computational cost and mapped data from proprietary germplasms may carry regulatory or intellectual property constraints. Construct-centric mapping with a conventional control remains a practical and widely used alternative, particularly in high-throughput settings. While neither strategy is universally preferred, a joint-reference mapping offers a more complete and unified treatment of alignment ambiguity and may be the natural choice for academic or smaller-scale workflows, and construct-centric mapping remains practical where computational efficiency, data volume, or germplasm confidentiality are constraining factors.

Forusers seeking to extend this workflow, the alignment strategy and reference configuration choices described above can feed directly into downstream or parallel workstreams. For example, an intuitive next step could be structural variant callers that use split-read and discordant-pair evidence to automate breakpoint detection (as demonstrated by Schouten et al. [21] who applied DELLY [45] to characterize T-DNA insertions in re-sequenced transgenic plants), or downstream assembly and variant calling for determining insert site integrity. Regardless, the read-mapping signatures illustrated here remain the interpretive foundation for validating and contextualizing any automated caller output. As each step in this analysis, from reference construction and alignment through BAM processing, coverage calculation, and junction review, is independently substitutable, the workflow is readily formalized using pipeline management systems such as Snakemake [46] or Nextflow [47] and scaled to meet the experimental and operational demands of any investigator.

## Conclusions

Whole genome sequencing has rapidly evolved into the regulatory standard for the molecular characterization of transgenic events. Since its initial regulatory acceptance in 2012, and following Canada’s guidance in 2017 and EFSA’s Technical Note in 2018 (updated in 2024), WGS has expanded substantially across major agricultural biotechnology markets, with growing adoption among research groups [6, 8–10, 12–21]. WGS-based pipelines allow for high-throughput characterization with nucleotide-level precision while eliminating the need for custom reagents required by Southern blotting and directed amplicon sequencing. This automation-ready approach can, in most cases, reliably deliver all regulatory endpoints for transgenic events in a single standardized workflow. With sequencing costs declining and algorithms improving, WGS-based characterization has become the preferred methodology in both research and regulatory settings.

Although this study focused on paired-end Illumina data for construct-based transformations, the interpretive framework extends broadly. The approach can be readily adapted to characterize site-directed modifications, such as those introduced via TALEN or CRISPR/Cas systems, as demonstrated by genome-wide WGS assessments showing high specificity and negligible off-target effects beyond background variation [48–50]. Short-read paired-end sequencing can in some cases face challenges in resolving highly repetitive inserts, complex integration sites, or large genomic rearrangements. Long-read sequencing platforms such as PacBio and Oxford Nanopore offer promising opportunities to complement or replace short-read approaches in these cases, providing improved resolution for complex structures and repetitive regions [51–53]. As these technologies become more cost-effective and accessible, hybrid and long-read-only workflows are increasingly being integrated into comprehensive molecular characterization pipelines, expanding the resolving power for increasingly complex or stacked traits.

The framework presented here provides a robust, platform-agnostic foundation for WGS-based molecular characterization. The interpretive approach relies on fundamental sequencing outcomes including junction signatures, coverage patterns, and mate-pair behaviors that manifest consistently regardless of specific software choices. This toolkit-independent framework enables reliable interpretation across diverse transformation outcomes, provided that analysts understand the underlying read behaviors, data characteristics, and their experimental inputs. By focusing on observable signatures rather than prescribing specific tools, this approach accommodates the evolving landscape of alignment algorithms and visualization platforms while maintaining interpretive consistency.

Currently, regulatory momentum has outpaced the development of broadly accessible interpretation frameworks for WGS-based molecular characterization. The read-mapping signatures and control strategies presented here address this gap, providing practical guidance for laboratories implementing WGS-based characterization and regulators interpreting submission data. The figures and workflows represent a core set of expectations and common observations encountered during paired-end sequencing analysis. To streamline the process, investigators should develop strong working knowledge of transformation construct components and typical T-DNA integration signatures. Awareness of common construct elements (host organism endogenous sequences, duplicated T-DNA/backbone regions, etc.) aids in both validating expected transformation outcomes and recognizing deviations.

This work establishes a foundation for consistent, transparent evaluation of transgenic events by defining clear interpretive signatures and providing representative examples across diverse transformation outcomes. The focus on observable mapping signatures rather than specific software ensures robust molecular characterization using widely available WGS tools while maintaining flexibility as technologies evolve. These core principles provide a framework for ensuring accuracy, reproducibility, and confidence in WGS-based molecular characterizations as the field continues to advance.

## Electronic supplementary material

Below is the link to the electronic supplementary material.

Supplementary material 1

## Data Availability

This study uses simulated sequencing data, supplemented by one real-world dataset (MON 89788). All simulation reference sequences were synthetically generated using scripts available as part of the BBTools package and as described in Materials and Methods. The complete simulation supporting dataset, including simulated sequencing reads (FASTQ), synthetic reference sequences (FASTA), alignment files (BAM), junction and flanking sequence analyses, and scripts used for generating inserts and reads, is publicly available through figshare (https://doi.org/10.6084/M9.FIGSHARE.30920966) [30]. For the real-world example (Fig. 9, S10A Fig, S10B Fig), alignment files (BAM) for MON 89788 mapped against the proprietary transformation construct and against the soybean *Le1* locus (GenBank K00821.1) are included in the same figshare deposit. The proprietary transformation construct reference sequence and raw FASTQ reads are not publicly available due to intellectual property restrictions; however, the T-DNA portion of the MON 89788 insert is publicly available under GenBank accession HW805122.1, which is sufficient to reproduce the T-DNA region mapping shown in Fig 9 from any independently generated MON 89788 read source.

## Abbreviations

WGS: Whole genome sequencing
T-DNA: Transfer DNA
SCL: Single-copy locus
EFSA: European Food Safety Authority
CFIA: Canadian Food Inspection Agency
MFDS: Ministry of Food and Drug Safety (Korea)
FDA: Food and Drug Administration
IGV: Integrative Genomics Viewer
SNP: Single nucleotide polymorphism
bp: Base pair
kb: Kilobase
Mb: Megabase
LR: Left-Right (read orientation)
RL: Right-Left (read orientation)
LL: Left-Left (read orientation)
RR: Right-Right (read orientation)
BAM: Binary Alignment Map
