# Supplementary material for: Read-mapping signatures for molecular characterization of transgenic DNA insertions in paired-end Illumina data
Source: BMC Biotechnol. 2026 Jun 26;26:118. doi: 10.1186/s12896-026-01185-3 (PMC13651185; doi:10.1186/s12896-026-01185-3)
Supplement: Supplementary file 1 [file 12896_2026_1185_MOESM1_ESM.docx]

# Supporting Information

## Supplement to Read-Mapping Signatures for Molecular Characterization of Transgenic DNA Insertions in Paired-End Illumina Data

Colton R. Kessenich^1*^, Nicole Catherall^1^, Elisa Hughes^1^, Kyle Skottke^1^, Jason Vest^1^, Kristen E. Wendt^1^

^1^Bayer Crop Science, 700 Chesterfield Pkwy W, Chesterfield, MO, 63017, USA

^*^Correspondence: colton.kessenich@bayer.com

| S1 Table. Summary of Examples and Interpretive Signatures | | |
| --- | --- | --- |
| Figure | Example | Core Features |
| Fig 1 | Transformation construct map | Reference vector used in simulations; shows intended T-DNA vs. backbone regions. |
| Fig 2 | Five mapped-read categories | Schematic of five informative read categories defining WGS junction signatures. |
| Fig 3 | Single-copy T-DNA insert | Uniform coverage across T-DNA with two distinct border junctions; no backbone reads. |
| Fig 4 | Multiple insertions | Two sets of junctions establish two independent insertions; T-DNA coverage consistent with that interpretation. |
| Fig 5 | Common backbone element | Pyramidal coverage over a duplicated promoter in the backbone without junction reads. |
| Fig 6 | Endogenous homology in T-DNA | Elevated coverage and junction-like signals over a promoter that is endogenous to native genome. |
| Fig 7 | Control mapping (homologous feature) | Negative-control mapping reproduces the promoter signature, proving it’s endogenous. |
| Fig 8 | Integrated example event | Single-copy insert; low-depth reads over ori indicate contamination; duplicated promoter coverage. |
| Fig 9 | Real‑world example | Single‑copy T‑DNA insertion exhibiting canonical junction pileups and uniform coverage, with read‑mapping signatures consistent with simulated single‑copy examples. |
| S1 Fig | Positive control | Confirms uniform sequencability; expected junction-like artifacts at linearization ends. |
| S2A Fig | Negative control (no homology) | Lack of control reads mapping to construct elements, no endogenous homology. |
| S2B Fig (Fig7) | Negative control (homologous feature) | Control reads map to construct element, endogenous homology to discount. |
| S3 Fig | Endogenous single copy locus control | Coverage over SCL establishes empirical sequencing depth baseline for the dataset. |
| S4 Fig | T-DNA + backbone at one locus | Intended T-DNA plus adjacent backbone inserted together; single-copy coverage. |
| S5 Fig | Backbone at separate locus | Standard T-DNA plus backbone-only insert at a different genomic locus. |
| S6 Fig | Indels in T-DNA | Standard insert with localized in-dels; confirm via consensus and alignment. |
| S7 Fig | T-DNA tandem co-fragment | Fragmented portion of T-DNA co inserted at flank |
| S8 Fig | Construct contamination | Consistent coverage across non-insert construct regions, library carryover. |
| S9 Fig | Bacterial contamination | Non genomic equivalent mapping with sporadic reads and mismatches localized to bacterial element(s). |
| S10A Fig | Real-data negative control mapping | Lack of control reads mapping to construct elements, no endogenous homology. |
| S10B Fig | Real-data single-copy endogenous locus mapping | Coverage over SCL establishes empirical sequencing depth baseline for the dataset. |
| S11 Data | Single‑copy junction sequences | Complete alignment showing construct-to-genome transition (^) and progressive truncation defining boundaries; consensus extraction yields flanking sequences |
| S12 Data | Multiple insertion junction sequences | Four distinct junction alignments with unique flanks; consensus sequences enable chromosomal disambiguation |
| S13 Data | Chromosomal localization of insertions | FASTA alignments of consensus flanks to chromosome confirm independent insertion sites and enable junction pairing. |

**S1 Fig. Positive control: mapping the construct against itself to demonstrate sequencability.** Reads from the transformation construct mapped back to the construct confirm uniform sequencability across the plasmid. Junction-like signals at the linearized ends and RL orientation reads (green) are expected artifacts of mapping a circular plasmid as linear; these do not represent structural variation. Coverage should be relatively uniform at the achieved depth (e.g., ~75×).

**S2A Fig. Negative control showing absence of endogenous homology to construct sequences.** Reads from the conventional control do not map to specific construct elements, indicating no endogenous sequence homology that would need to be accounted for when interpreting transformed-material mappings. This result validates that observed construct-mapping reads in transgenic samples represent true DNA insertions.

**S2B Fig. Negative control with construct-mapping reads indicating endogenous sequence homology.** Reads mapping from the conventional control to specific construct elements indicate endogenous homology between the host genome and transformation construct. Comparable pileups in the transformed material should be attributed to this endogenous homology rather than transgene insertion. This negative control mapping is essential for accurate interpretation and is shown as Fig 7 in the main manuscript.

**S3 Fig. Single-copy endogenous locus control establishing empirical genome-wide sequencing depth.** Mapping to a single-copy endogenous locus (SCL) provides an empirical reference for genome-wide depth (e.g., ~75×) against which T-DNA coverage observations can be compared as a consistency check alongside junction analysis. Junction-like signals of unpaired reads at the locus ends reflect sequence boundaries of the reference, not potential insertions.

**S4 Fig. Single-locus co-integration of T-DNA and contiguous backbone fragment.** The intended T-DNA is present with one junction in the Left Border region and a second junction within the Maintenance Factor element of the backbone. Coverage across the combined T-DNA and adjacent backbone region is visually determined to be similar to that of the computed single-copy locus. No reads map to the remaining backbone. Interpretation: a single insertion containing T-DNA plus adjacent backbone sequence.

**S5 Fig. Two independent insertions: complete T-DNA plus backbone fragment at separate loci.** Standard single-copy T-DNA (two junctions at borders) plus an additional backbone-only insert with its own pair of junctions at separate coordinates. The coverage across both inserts is similar to that of the computed single-copy coverage. Interpretation: co-inserted backbone fragment at a distinct genomic locus; characterize each insert independently.

**S6A Fig.** **Standard single-copy T-DNA with sequence polymorphisms.** At this broad scale, the insert appears as a standard single-copy event, though subtle sequence-based differences are present. Single nucleotide polymorphisms (SNPs) appear as consistent alternate base colors (e.g., T > G shown in red). Insertions appear as purple "I" indicators (solid purple bar when zoomed out). Deletions show black lines in the read data and coverage dips or holes that may be difficult to discern at this view. Large polymorphisms may be visible when scaled out, but small deviations require closer inspection (S6B Fig.).

**S6B Fig. Magnified view revealing SNPs, insertions, and deletions not visible at broad scale.** At this magnified scale, polymorphisms become clear. An SNP (T > G) appears as a red bar in the depth graph and consistent red bases in reads (enhancer region shown). Insertions are marked with purple "I" indicators. Deletions show reads punctured by black lines and clear coverage holes. Interpretation (applies to both S6A and S6B): Minor sequence differences relative to the reference construct; confirm via consensus extraction and pairwise alignment.

**S7 Fig. Complex insertion with co-integrated T-DNA fragment producing multiple chimeric pileups.** Elevated coverage and abnormal mate-pair spacing are observed at the left junction. Soft-clipped reads at the junction display two separate sets of flanking sequence patterns. Mate pairs across this region show anomalous spacings (extended or compressed insert sizes relative to expectation from size selection, which may result in discordant orientations depending on scope and scale of rearrangement; see two overlapped pairs at top panel). Additional chimeric read pileups occur within the T-DNA interior, uniquely paired with one junction pattern. Interpretation: a T-DNA fragment was co-inserted in tandem at the flank of the intact T-DNA. Junction sequences from each pileup should be extracted and aligned to validate the authentic genomic flank versus the internal T-DNA-to-T-DNA junction.

**S8 Fig. Construct contamination showing uniform low coverage across backbone without junction signatures.** A true single-copy T-DNA insert is present as expected, but note the additional low, uniform coverage across the non-T-DNA portions of the construct without discrete junctions; this often arises from library carryover. Interpretation: sequencing library is contaminated by the transformation construct; re-make libraries if contamination approaches experimental depth or confounds interpretation in any way.

**S9 Fig. Bacterial contamination showing sporadic ori coverage with sequence mismatches and truncations.** Paired reads confirm the intended T-DNA insert with clear unique junctions, and coverage is in line with expectation across the insert. Additional low-depth (~3×) coverage across the backbone ori region (bacterial derived element), along with sporadic truncated reads and sequence mismatches, are also present. Interpretation: minor bacterial contamination likely introduced during library preparation.

**S10A Fig. Real-data negative control mapping demonstrating absence of spurious construct mapping in a commercial soybean genome.** Whole-genome Illumina paired-end reads from the conventional non-transformed parent line of Roundup Ready 2 Yield soybean (MON 89788) mapped to the transformation construct used for event generation. No reads map to T-DNA or backbone regions of the construct, indicating no endogenous sequence homology between the host genome and construct elements under standard library preparation and mapping conditions. This result validates that construct-mapping reads observed in the transformed sample represent genuine T-DNA insertion rather than background or spurious alignment, consistent with the simulated negative control shown in S2A Fig. Interpretation: no endogenous homology to the transformation construct; construct-mapping reads in the transformed material can be attributed to true insertion.

**S10B Fig. Real-data single-copy endogenous locus mapping establishing empirical sequencing depth for the MON89788 dataset.** Whole-genome Illumina paired-end reads from Roundup Ready 2 Yield soybean (MON 89788) mapped to the soybean lectin gene locus *Le1* (GenBank K00821.1), consistent with the single-copy endogenous reference established by Kovalic et al. [20]. Calculated coverage across the locus averaged 150.125×, establishing the empirical depth baseline for interpreting T-DNA coverage in Fig 9. The observed localized coverage gaps reflect single nucleotide deletions in the test material germplasm relative to reference sequence and do not meaningfully affect the overall depth estimate. Interpretation: empirical sequencing depth of ~150x successfully established; *Le1* coverage provides the baseline reference for the accompanying real-data characterization.

**S11 Data. Junction-spanning read alignments from single-copy insertion event.**

Multiple sequence alignment of chimeric reads extracted from left border and right border junction pileups. The construct-to-genome transition is marked with ^. Reads spanning up to the junction confirm the construct sequence, while progressively truncated reads (soft-clipped portions colored to match IGV appearance) extend into the flanking genomic DNA, defining the precise junction boundary at a single consistent coordinate. Consensus sequences of the unaligned portions yield the flanking genomic sequence (shown in bold at bottom of each alignment). Mapping these consensus flanking sequences to a host genome reference allows determination of the chromosomal localization of the insertion.

**Left Border**

-----TACCATCCTTCGATTCGTGGCTGTAGATAGATCTTAAATAGTTCACTTATACCTTGGATTTAACGGCCGATTCTAAAACACTTCGAGGGCTAGTCAAGGAGGCCATACTAACC**^**AATATGAGAGGCCGTATCTCGGTATCTGAATCCTCTC------------------------------------------------------------------------------------------------------------------------

-----------------ATTCGTGGCTGTAGATAGATCTTAAATAGTTCACTTATACCTTGGATTTAACGGCCGATTCTAAAACACTTCGAGGGCTAGTCAAGGAGGCCATACTAACC**^**AATATGAGAGGCCGTATCTCGGTATCTGAATCCTCTCGCTCATTGACCT------------------------------------------------------------------------------------------------------------

----------TCCTTCGATTCGTGGCTGTAGATAGATCTTAAATAGTTCACTTATACCTTGGATTTAACGGCCGATTCTAAAACACTTCGAGGGCTAGTCAAGGAGGCCATACTAACC**^**AATATGAGAGGCCGTATCTCGGTATCTGAATCCTCTCGCTCA-------------------------------------------------------------------------------------------------------------------

----------------GATTCGTGGCTGTAGATAGATCTTAAATAGTTCACTTATACCTTGGATTTAACGGCCGATTCTAAAACACTTCGAGGGCTAGTCAAGGAGGCCATACTAACC**^**AATATGAGAGGCCGTATCTCGGTATCTGAATCCTCTCGCTCATTGACC-------------------------------------------------------------------------------------------------------------

ATAGATACCATCCTTCGATTCGTGGCTGTAGATAGATCTTAAATAGTTCACTTATACCTTGGATTTAACGGCCGATTCTAAAACACTTCGAGGGCTAGTCAAGGAGGCCATACTAACC**^**AATATGAGAGGCCGTATCTCGGTATCTGAATC-----------------------------------------------------------------------------------------------------------------------------

--------CATCCTTCGATTCGTGGCTGTAGATAGATCTTAAATAGTTCACTTATACCTTGGATTTAACGGCCGATTCTAAAACACTTCGAGGGCTAGTCAAGGAGGCCATACTAACC**^**AATATGAGAGGCCGTATCTCGGTATCTGAATCCTCTCTCT---------------------------------------------------------------------------------------------------------------------

---GATACCATCCTTCGATTCGTGGCTGTAGATAGATCTTAAATAGTTCACTTATACCTTGGATTTAACGGCCGATTCTAAAACACTTGGAGGGCTAGTCAAGGAGGCCATACTAACC**^**AATATGAGAGGCCGTATCTCGGTATCTGAATCCTC--------------------------------------------------------------------------------------------------------------------------

----------TCCTTCGATTGGTGGATGTAGATAGATCTTAAATAGTTCACTTATACCTTGGATTTAACGGCCGATTCTAAAACACTTCGAGGGCTAGTCAAGGAGGCCATACTAACC**^**AATATGAGAGGCCGTATCTCGGTATCTGAATCCTCTCGCTCA-------------------------------------------------------------------------------------------------------------------

---------------------------------AGATCTTAAATAGTTCACTTATACCTTGGATTTAACGGCCGATTCTAAAACACTTCGAGGGCTAGTCAAGGAGGCCATACTAACC**^**AATATGAGAGGCCGTATCTCGGTATCTGAATCCTCTCGCTCATTGACCTGTTATAACCATGGAAC--------------------------------------------------------------------------------------------

-----------------------------------------------------------------TAACGGCCGATTCTAAAACACTTCGAGGGCTAGTCAAGGAGGCCATACTAACC**^**AATATGAGAGGCCGTATCTCGGTATCTGAATCCTCTCGCTCATTGACCTGTTATAACCATGGAACTCTGGCGAGTCGGGGCGAATATGGACTATATT------------------------------------------------------------

----------------------------TAGATAGATCTTAAATAGTTCACTTATACCTTGGATTTAACGGCCGATTCTAAAACACTTCGAGGGCTAGTCAAGGAGGCCATACTAACC**^**AATATGAGAGGCCGTATCTCGGTATCTGAATCCTCTCGCTCATTGACCTGTTATAACCAT-------------------------------------------------------------------------------------------------

------------------------------------TCTTAAATAGTTCACTTATACCTTGGATTTAACGGCCGATTCTAAAACACTTCGAGGGCTAGTCAAGGAGGCCATACTAACC**^**AATATGAGAGGCCGTATCTCGGTATCTGAATCCTCTCGCTCATTGACCTGTTATAACCATGGAACTCT-----------------------------------------------------------------------------------------

------------------------------------------------------------------AAAGGCCGATTCTAAAACACTTCGAGGGCTAGTCAAGGAGGCCATACTAACC**^**AATATGAGAGGCCGTATCTCGGTATCTGAATCCTCTCGCTCATTGACCTGTTATAACCATGGAACTCTGGCGAGTCGGGGCGAATATGGACTATATTT-----------------------------------------------------------

-----------------------------------------------TCACTTATACCTTGGATTTAACGGCCGATTCTAAAACACTTCGAGGGCTAGTCAAGGAGGCCATACTAACC**^**AATATGAGAGGCCGTATCTCGGTATCTGAATCCTCTCGCTCATTGACCTGTTATAACCATGGAACTCTGGCGAGTCGGG------------------------------------------------------------------------------

------------------------------------TCTTAAATAGTTCACTTATACCTTGGATTTAACGGCCGATTCTAAAACACTTCGAGGGCTAGTCAAGGAGGCCATACTAACC**^**AATATGAGAGGCCGTATCTCGGTATCTGAATCCTCTCGCTCATTGACCTGTTATAACCATGGAACTCT-----------------------------------------------------------------------------------------

--------------------------------------------AGTTCACTTATACCTTGGATTTAACGGCCGATTCTAAAACACTTCGAGGGCTAGTCAACGAGGCCGTACTAACC**^**AATATGAGAGGCCGTATCTCGGTATCTGAATCCTCTCGCTCATTGACCTGTTATAACCATGGAACTCTGGCGAGTC---------------------------------------------------------------------------------

-------------------TCGTGGCTGTAGATAGATCTTAAATAGTTCACTTATACCTTGGATTTAACGGCCGATTCTAAAACACTTCGAGGGCTAGTCAAGGAGGCCATACTAACC**^**AATATGAGAGGCCGTATCTCGGTATCTGAATCCTCTCGCTCATTGACCTGT----------------------------------------------------------------------------------------------------------

-----------------------------------------------TCACTTATACCTTGGATTTAACGGCCGATTCTAAAACACTTCGAGGGCTAGTCAAGGAGGCCATACTAACC**^**AATATGAGAGGCCGTATCTCGGTATCTGAATCCTCTCGCTCATTGACCTGTTATAACCATGGAACTCTGGCGAGTCGGG------------------------------------------------------------------------------

------------------------------------------------------TACCTTGGATTTAACGGCCGATTCTAAAACACTTCGAGGGCTAGTCAAGGAGGCCATACTAACC**^**AATATGAGAGGCCGTATCTCGGTATCTGAATCCTCTCGCTCATTGACCTGTTATAACCATGGAACTCTGGCGAGTCGGGGCGAATA-----------------------------------------------------------------------

--------------------------------------------------CTTATACCTTGGATTTAACGGCCGATTCTAAAACACTTCGAGGGCTAGTCAAGGAGGCCATACTAACC**^**AATATGAGAGGCCGTATCTCGGTATCTGAATCCTCTCGCTCATTGACCTGTTATAACCATGGAACTCTGGCGAGTCGGGGCG---------------------------------------------------------------------------

-----------------------------------------------------ATACCTTGGATTTAACGGCCGATTCTAAAACACTTCGAGGGCTAGTCAAGGAGGCCATACTAACC**^**AATATGAGAGGCCGTATCTCGGTATCTGAATCCTCTCGCTCATTGACCTGTTATAACCATGGAACTCTGGCGAGTCGGGGCGAAT------------------------------------------------------------------------

-----------------------GGCTGTAGATAGATCTTAAATAGTTCACTTATACCTTGGATTTAACGGCCGATTCTAAAACACTTCGAGGGCTAGTCAAGGAGGCCATACTAACC**^**AATATGAGAGGCCGTATCTCGGTATCTGAATCCTCTCGCTCATTGACCTGTTATA------------------------------------------------------------------------------------------------------

-------------------------------------------------------------GATTTAACGGCCGATTCTAAAACACTTCGAGGGCTAGTCAAGGAGGCCATACTAACC**^**AATATGAGAGGCCGTATCTCGGTATCTGAATCCTCTCGCTCATTGACCTGTTATAACCATGGAACTCTGGCGAGTCGGGGCGAATATGGACTA----------------------------------------------------------------

-------------------------------------------------------ACCTTGGATTTAACGGCCGATTCTAAAACACTTCGAGGGCTAGTCAAGGAGGCCATACTAACC**^**AATAGGAGAGGCCGTATCTCGGTATCTGAATCCTCTCGCTCATTGACCTGTTATAACCATGGAACTCTGGCGAGTCGGGGCGAATAT----------------------------------------------------------------------

-------------------------------ATAGATCTTAAATAGTTCACTTATACCTTGGATTTAACGGCCGATTCTAAAACACTTCGAGGGCTAGTCAAGGAGGCCATACTAACC**^**AATATGAGAGGCCGTATCTCGGTATCTGAATCCTCTCGCTCATTGACCTGTTATAACCATGGA----------------------------------------------------------------------------------------------

----------------------------------------------------------------------------TCTAAAACACTTCGAGGGCTAGTCAAGGAGGCCATACTAACC**^**AATATGAGAGGCCGTATCTCGGTATCTGAATCCTCTCGCTCATTGACCTGTTATAACCATGGAACTCTGGCGAGTCGGGGCGAATATGGACTATATTTAGCTCTCATG-------------------------------------------------

-----------------------------------------------------------------------CCGATTCTAAAACACTTCGAGGGCTAGTCAAGGAGGCCATACTAACC**^**AATATGAGAGGCCGTATCTCGGTATCTGAATCCTCTCGCTCATTGACCTGTTATAACCATGGAACTCTGGCGAGTCCGGGCGAATATGGACTATATTTAGCTC------------------------------------------------------

----------------------------------------------------------------------------------------------------------------CTAACC**^**AATATGAGAGGCCGTATCTCGGTATCTGAATCCTCTCGCTCATTGACCTGTTATAACCATGGAACTCTGGCGAGTCGGGGCGAATATGGACTATATTTAGCTCTCATGCGTGTTATATGTATTGCATCATGTCAAATTTACACC-------------

---------------------------------------------------------------------------------------TCGAGGGCTAGTCAAGGAGGCCATACTAACC**^**AATATGAGAGGCCGTATCTCGGTATCTGAATCCTCTCGCTCATTGACCTGTTATAACCATGGAACTCTGGCGAGTCGGGGCGAATATGGACTATATTTAGCTCTCATGCGTGTTATATG--------------------------------------

-----------------------------------------------------------------------------------------------TAGTCAAGGAGGCCATACTAACC**^**AATATGAGAGGCCGTATCTCGGTATCTGAATCCTCTCGCTCATTGACCTGTTATAACCATGGAACTCTGGCGAGTCGGGGCGAATATGGACTATATTTAGCTCTCATGCGTGTTATATGGATTGCAT------------------------------

----------------------------------------------------------------------------------------------CTAGTCAAGGAGGCCATACTAACC**^**AATATGAGAGGCCGTATCTCGGTATCTGAATCCTCTCGCTCATTGACCTGTTATAACCATGGAACTCTGGCGAGTCGGGGCGAATATGGACTATATTTAGCTCTCATGCGTGTTATATGTATTGCA-------------------------------

--------------------------------------------------------------------------ATTCTAAAACACTTCGAGGGCTAGTCAAGGAGGCCATACTAACC**^**AATATGAGAGGCCGTATCTCGGTATCTGAATCCTCTCGCTCATTGACCTGTTATAACCACGGAACTCTGGCGAGTCGGGGCGAATATGGACTATATTTAGCTCTCA---------------------------------------------------

---------------------------------------------------------------------------------------------------------GGCCATACTAACC**^**AATATGAGAGGCCGTATCTCGGTATCTGAATCCTCTCGCTCATTGACCTGTTATAACCATGGAACTCTGGCGAGTCGGGGCGAATATGGACTATATTTAGCTCTCATGCGTGTTATATGTATTGCATCATGTCAAAT--------------------

--------------------------------------------------------------------CGGCCGATTCTAAAACACTTCGAGGGCTAGTCAAGGAGGCCATACTAACC**^**AATATGAGAGGCCGTATCTCGGTATCTGAATCCTCTCGCTCATTGACCTGTTATAACCATGGAACTCTGGCGAGTCGGGGCGAATATGGACTATATTTAG---------------------------------------------------------

-------------------------------------------------------------------------------------------GGGCTAGTCAAGGAGGCCATACTAACC**^**AATATGAGAGGCCGTATCTCGGTATCTGAATCCTCTCGCTCATTGACCTGTTATAACCATGGAACTCTGGCGAGTCGGGGCGAATATGGACTATATTTAGCTCTCATGCGTGTTATATGTATT----------------------------------

---------------------------------------------------------------------------TTCTAAAACACTTCGAGGGCTAGTCAAGGAGGCCATACTAACC**^**AATATGAGAGGCCGTATCTCGGTATCTGAATCCTCTCGCTCATTGACCTGTTATAACCATGGAACTCTGGCGAGTCGGGGCGAATATGGACTATATTTAGCTCTCAT--------------------------------------------------

----------------------------------------------------------------------------------------------------------------------**^**AATATGAGAGGCCGTATCTCGGTATCTGAATCCTCTCGCTCATTGACCTGTTATAACCATGGAACTCTGGCGAGTCGGGGCGAATATGGACTATATTTAGCTCTCATGCGTGTTATATGTATTGCATCATGTCAAATTTACACCAGTTAA-------

---------------------------------------------------------------------------------------------GCTAGTCAAGGAGGCCATACTAACC**^**AATATGAGAGGCCGTATCTCGGTATCTGAATCCTCTCGCTCATTGACCTGTTATAACCATGGAACTCTGGCGAGTCGGGGCGAATATGGACTATATTTAGCTCTCATGCGTGTTATATGTATTGC--------------------------------

--------------------------------------------------------------------------------------------------------------------CC**^**AATATGAGAGGCCGTATCTCGGTATCTGAATCCTCTCGCTCATTGACCTGTTATAACCATGGAACTCTGGCGAGTCGGGGCGAATATGGACTATATTTAGCTCTCATGCGTGTTATATGTATTGCATCATGTCAAATTTACACCAGTT---------

---------------------------------------------------------------------------------------------------------------ACTAACC**^**AATATGAGAGGCCGTATCTCGGTATCTGAATCCTCTCGCTCATTGACCTGTTATAACCATGGAACTCTGGCGAGTCGGGGCGAATATGGACTATATTTAGCTCTCATGCGTGTTATATGTATTGCATCATGTCAAATTTACGC--------------

------------------------------------------------------------------------------------------------AGTCAAGGAGGCCATACTAACC**^**AATATGAGAGGCCGTATCTCGGTATCTGAATCCTCTCGCTCATTGACCTGTTATAACCATGGAACTCTGGCGAGTCGGGGCGAATATGGACTATATTTAGCTCTCATGCGTGTTATATGTATTGCATC-----------------------------

----------------------------------------------------------------------------------------------------------------------**^**-ATATGAGAGGCCGTATCTCGGTATCTGAATCCTCTCGCTCATTGACCTGTTATAACCATGGAACTCTGGCGAGTCGGGGCGAATATGGACTATATTTAGCTCTCATGCGTGTTATATGTATTGCATCATGTCAAATTTACACCAGTTAAA------

----------------------------------------------------------------------------------------------------------------------**^**-ATATGAGAGGCCGTATCTCGGTATCTGAATCCTCTCGCTCATTGACCTGTTATAACCATGGAACTCTGGCGAGTCGGGGCGAATATGGACTATATTTAGCTCTCATGCGTGTTATATGTATTGCATCATGTCAAATTTACACCAGTTAAA------

----------------------------------------------------------------------------------------------------------------------**^**-ATATGAGAGGCCGTATCTCGGTATCTGAATCCTCTCGCTCATTGACCTGTTATAACCATGGAACTCTGGCGAGTCGGGGCGAATATGGACTATATTTAGCTCTCATGCGTGTTATATGTATTGCATCATGTCAAATTTACACCAGTTAAA------

----------------------------------------------------------------------------------------------------------------------**^**---ATGAGAGGCCGTATCTCGGTATCTGAATCCTCTCGCTCATTGACCTGTTATAACCATGGAACTCTGGCGAGTCGGGGCGAATATGGACTATATTTAGCTCTCATGCGTGTTATATGTATTGCATCATGTCAAAATTACACCAGTCAAAGG----

----------------------------------------------------------------------------------------------------------------------**^**------AGAGGCCGTATCTCGGTATCTGAATCCTCTCGCTCATTGACCTGTTATAACCATGGAACTCTGGCGAGTCGGGGCGAATATGAACTATATTTAGCTCTCATGCGTGTTATATGTATTGCATCATGTCAAATTTACACCAGTTAAAGGGAG-

----------------------------------------------------------------------------------------------------------------------**^**-------GAGGCCGTATCTCGGTATCTGAATCCTCTCGCTCATTGACCTGTTATAACCATGGAACTCTGGCGAGTCGGGGCGAATATGGACTATATTTAGCTCTCATGCGTGTTATATGTATTGCATCATGTCAAATTTACACCAGTTAAAGGGAGA

----------------------------------------------------------------------------------------------------------------------**^**-------GAGGCCGTATCTCGGTATCTGAATCCTCTCGCTCATTGACCTGTTATAACCATGGAACTCTGGCGAGTCGGGGCGAATATGGACTATATTTAGCTCTCATGCGTGTTATATGTATTGCATCATGTCAAATTTACACCAGTTAAAGGGAGA

**---GATACCATCCTTCGATTCGTGGCTGTAGATAGATCTTAAATAGTTCACTTATACCTTGGATTTAACGGCCGATTCTAAAACACTTCGAGGGCTAGTCAAGGAGGCCATACTAACC^AATATGAGAGGCCGTATCTCGGTATCTGAATCCTCTCGCTCATTGACCTGTTATAACCATGGAACTCTGGCGAGTCGGGGCGAATATGGACTATATTTAGCTCTCATGCGTGTTATATGTATTGCATCATGTCAAATTTACACCAGTTAAAGGGAGA**

**Right Border**

-------------------------------------------------------------------------------------------------------------CCCGCACACTGAAGCCGCCCGATGGTATAAATAAAATCATGTGGATGCA**^**TTTTGCGCCGAGTGGACACTAGAGTAGGGGGACAAATCGGTACCCATTCCAGCAAAAAGTCGAAGAATACTCTGCTGCAGCCCGCAAGAAGAGAGGACCAC----------------

----------------------------------------------------------------------------------------------------------------GCACACTGAAGCCGCCCGATGGTATAAATAAAATCATGTGGATGCA**^**TTTTGCGCCGAGTGGACACTAGAGTAGGGGGACAAATCGGTACCCATTCCAGCAAAAAGTCGAAGAATACTCTGCTGCAGCCCGCAAGAAGAGAGGACTACATG-------------

------------------------------------------------------------------------------------------------------------------ACACTGAAGCCGCCCGATGGTATAAATAAAATCATGTGGATGCA**^**TTTTGCGCCGAGTGGACACTAGAGTAGGGGGACAAATCGGTACCCATTCCAGCAAAAAGTCGAAGAATACTCTGCTGCAGCCCGCAAGAAGAGAGGACCACATGAT-----------

-----------------------------------------------------------------------------------------------------------------------------GCCCGATGGTATAAATAAAATCATGTGGATGCA**^**TTTTGCGCCGAGTGGACACTAGAGTAGGGGGACAAATCGGTACCCATTCCAGCAAAAAGTCGAAGAATACTCTGCTGCAGCCCGCAAGAAGAGAGGACCACATGATTATCTGTTGAT

------------------------------------------------------------CTGCCCCTGACTCGGCTATCGGGTAGTATTAGCAAAAGGAGATTTTCGACCCGCACACTGAAGCCGCCCGATGGTATAAATAAAATCATGTGGATGCA**^**TTTTGCGCCGAGTGGACACTAGAGTAGGGGGACAAATCGGTACCCATTCCAG-----------------------------------------------------------------

-------------------------------------------------------------TGCCCCTGACTCGGCTATCGGGTAGCATTAGCAAAAGGAGATTTTCGACCCGCACACTGAAGCCGCCCGATGGTATAAATAAAATCATGTGGATGCA**^**TTTTGCGCCGAGTGGACACTAGAGTAGGGGGACAAATCGGTACCCATTCCAGC----------------------------------------------------------------

-------------------------------------------------------------TGCCCCTGACTCGGCTATCGGGTAGCATTAGCAAAAGGAGATTTTCGACCCGCACACTGAAGCCGCCCGATGGTATAAATAAAATCATGTGGATGCA**^**TTTTGCGCCGAGTGGACACTAGAGTAGGGGGACAAATCGGTACCCATTCCAGC----------------------------------------------------------------

--------------------------------------------------------------------GACTCGGCTATCGGGTAGCATTAGCAAAAGGAGATTTTCGACCCGCACACTGAAGCCGCCCGATGGTATAAATAAAATCATGTGGATGCA**^**TTTTGCGCCGAGTGGACACTAGAGTAGGGGGACAAATCGGTACCCATTCCAGCAAAAAGT---------------------------------------------------------

---------------------------------------------------------------------ACTCGGCTATCGGGTAGCATTAGCAAAAGGAGATTTTCGACCCGCACACTGAAGCCGCCCGATGGTATAAATAAAATCATGTGGATGCA**^**TTTTGCGCCGAGTGGACACTAGAGTAGGGGGACAAATCGGTACCCATTCCAGCAAAAAGTC--------------------------------------------------------

----------------------------------------------------------------------CTCGGCTATCGGGTAGCATTAGCAAAAGGAGATTTTCGACCCGCACACTGAAGCCGCCCGATGGTATAAATAAAATCATGTGGATGCA**^**TTTTGCGCCGAGTGGACACTAGAGTAGGGGGACAAATCGGTACCCATTCCAGCAAAAAGTCG-------------------------------------------------------

----------------------------------------------------------------------CTCGGCTATCGGGTAGCATTAGCAAAAGGAGATTTTCGACCCGCACACTGAAGCCGCCCGATGGTATAAATAAAATCATGTGGATGCA**^**TTTTGCGCCGAGTGGACACTAGAGTAGGGGGACAAATCGGTACCCATTCCAGCAAAAAGTCG-------------------------------------------------------

-----------------------------------------------------------------------TCGGCTATCGGGTAGCATTAGCAAAAGGAGATTTTCGACCCGCACACTGAAGCCGCCCGATGGTATAAATAAAATCATGTGGATGCA**^**TTTTGCGCCGAGTGGACACTAGAGTAGGGGGACAAATCGGTACCCATTCCAGCAAAAAGTCGA------------------------------------------------------

-----------------------------------------------------------------------------ATCGGGTAGCATTAGCAAAAGGAGATTTTCGACCCGCACACTGAAGCCGCCCGATGGTATAAATAAAATCATGTGGATGCA**^**TTTTGCGCCGAGTGGACACTAGAGTAGGGGGACAAATCGGTACCCATTCCAGCAAAAAGTCGAAGAATA------------------------------------------------

--------------------------------------------------------------------------------GGGTAGCATTAGCAAAAGGAGATTTTCGACCCGCACACTGAAGCCGCCCGATGGTATAAATAAAATCATGTGGATGCA**^**TTTTGCGCCGAGTGGACACTAGAGTAGGGGGACAAATCGGTACCCATTCCAGCAAAAAGTCGAAGAATACTC---------------------------------------------

------------------------------------------------------------------------------------AGGATTAGCAAAAGGAGATTTTCGACCCGCACACTGAAGCCGCCCGATGGTATAAATAAAATCATGTGGATGCA**^**TTTTGCGCCGAGTGGACACTAGAGTAGGGGGACAAATCGGTACCCATTCCAGCAAAAAGTCGAAGAATACTCTGCT-----------------------------------------

------------------------------------------------------------------------------------AGCATTAGCAAAAGGAGATTTTCGACCCGCACACCGAAGCCGCCCGATGGTATAAATAAAATCATGTGGATGCA**^**TTTTGCGCCGAGTGGACACTAGAGTAGGGGGACAAATCGGTACCCATTCCAGCAAAAAGTCGAAGAATACTCTGCT-----------------------------------------

--------------------------------------------------------------------------------------CATTAGCAAAAGGAGATTTTCGACCCGCACACTGATGCCGCCCGATGGTATAAATAAAATCATGTGGATGCA**^**TTTTGCGCCGAGTGGACACTAGAGTAGGGGGACAAATCGGTACCCATTCCAGCAAAAAGTCGAAGAATACTCTGCTGC---------------------------------------

------------------------------------------------------------------------------------------AGCAAAAGGAGATTTTCGACCCGCACACTGAAGCCGCCCGATGGTATAAATAAAATCATGTGGATGCA**^**TTTTGCGCCGAGTGGACACTAGAGTAGGGGGACAAATCGGTACCCATTCCAGCAAAAAGTCGAAGAATACTCTGCTGCAGCC-----------------------------------

-----------------------------------------------------------------------------------------------AAGGAGATTTTCGACCCGCACACTGAAGCCGCCCGATGGTATAAATAAAATCATGTGGATGCA**^**TTTTGCGCCGAGTGGACACTAGAGTAGGGGGACAAATCGGTACCCATTCCAGCAAAAAGTCGAAGAATACTCTGCTGCAGCCCGCAA------------------------------

--------------------------------------------------------------------------------------------------GAGATTTTCGACCCGCACACTGAAGCCGCCCGATGGTATAAATAAAATCATGTGGATGCA**^**TTTTGCGCCGAGTGGACACTAGAGTAGGGGGACAAATCGGTACCCATTCCAGCAAAAAGTCGAAGAATACTCTGCTGCAGCCCGCAAGAA---------------------------

----------------------------------------------------------------------------------------------------------CGACCCGCACACTGAAGCCGCCCGATGGTATAAATAAAATCATGTGGATGCA**^**TTTTGCGCCGAGTGGACACTAGAGTAGGGGGACAAATCGGTACCCATTCCAGCAAAAAGTCGAAGAATACTCTGCTGCAGCCCGCAAGAAGAGAGGAC-------------------

ACCTACATCGATAATATCCCGAACGACCGCTGGCCCTGGACGGAAGCTAATGGACGGGCGCTGCCCCTGACTCGGCTATCGGGTAGCATTAGCAAAAGTAGATTTTCGACCCGCACACTGAAGCCGCCCGATGGTATA--------------------**^**---------------------------------------------------------------------------------------------------------------------

ACCTACATCGATAATATCCCGAACGACCGCTGGCCCTGGACGGAAGCTAATGGACGGGCGCTGCCCCTGACTCGGCTATCGGGTAGCATTAGCAAAAGGAGATTTTCGACCCGCACACTGAAGCCGCCCGATGGTATA--------------------**^**---------------------------------------------------------------------------------------------------------------------

ACCTACATCGATAATATCCCGAACGACCGCTGGCCCTGGACGGAAGCTAATGGACGGGCGCTGCCCCTGACTCGGCTATCGGGTAGCATTAGCAAAAGGAGATTTTCGACCCGCACACTGAAGCCGCCCGATGGTATAAATAA---------------**^**---------------------------------------------------------------------------------------------------------------------

ACCTACATCGATAATATCCCGAACGACCGCTGGCCCTGGACGGAAGCTAATGGACGGGCGCTGCCCCTGACTCGGCTATCGGGTAGCATTAGCAAAAGGAGATTTTCGACCCGCACACTGAAGCCGCCCGATGGTATAAATAAAATCA----------**^**---------------------------------------------------------------------------------------------------------------------

-CCTACATCGATAATATCCCGAACGACCGATGGCCCTGGACGGAAGCTAATGGACGGGCGCTGCCCCTGACTCGGCTATCGGGTAGCATTAGCAAAAGGAGATTTTCGACCCGCACACTGAAGCCGCCCGATGGTATAAATAAAATCATGT-------**^**---------------------------------------------------------------------------------------------------------------------

---------GATAATATCCCGAACGACCGCTGGCCCTGGACGGAAGCTAATGGACGGGCGCTGCCCCTGACTCGGCTATCGGGTAGCATTAGCAAAAGGAGATTTTCGACCCGCACACTGAAGCCGCCCGATGGTATAAATAAAATCATGTGGATGCA**^**T--------------------------------------------------------------------------------------------------------------------

---------GATAATATCCCGAACGACCGCTGGCCCTGGACGGAAGCTAATGGACGGGCGCTGCCCCTGACTCGGCTATCGGGTAGCATTAGCAAAAGGAGATTTTCGACCCGCACACTGAAGCCGCCCGATGGTATAAATAAAATCATGTGGATGCA**^**T--------------------------------------------------------------------------------------------------------------------

-----------TAATATCCCGAACGACCGCTGGCCCTGGACGGAAGCTAATGGACGGGCGCTGCCCCTGACTCGGCTATCGGGTAGCATTAGAAAAAGGAGATTTTCGACCCGCACACTGAAGCCGCCCGATGGTATAAATAAAATCATGTGGATGCA**^**TTT------------------------------------------------------------------------------------------------------------------

-----------------CCCGAACGACCGCTGGCCCTGGACGGAAGCTAATGGACGGGCGCTGCCCCTGACTCGGCTATCGGGTAGCATTAGCAAAAGGAGATTTTCGACCCGCACACTTAAGCCGCCCGATGGTATAAATAAAATCATGTGGATGCA**^**TTTTGCGCC------------------------------------------------------------------------------------------------------------

-----------------------CGACCGCTGGCCCTGGACGGAAGCTAATGGACGGGCGCTGCCCCTGACTCGGCTATCGGGTAGCATTAGCAAAAGGAGATTTTCGACCCGCACACTGAAGCCGCCCGATGGTATAAATAAAATCATGTGGATGCA**^**TTTTGCGCCGAGTGG------------------------------------------------------------------------------------------------------

------------------------GACCGCTGGCCCTGGACGGAAGCTAATGGACGGGCGCTGCCCCTGACTCGGCTATCGGGTAGCATTAGCAAAAGGAGATTTTCGACCCGCACACTGAAGCCGCCCGATGGTATAAATAAAATCATGTGGATGCA**^**TTTTGCGCCGAGTGGA-----------------------------------------------------------------------------------------------------

--------------------------CCGCTGGCCCTGGACGGAAGCTAATGGACGGGCGCTGCCCCTGACTCGGCTATCGGGTAGCATTAGCAAAAGGAGATTTTCGACCCGCACACTGAAGCCGCCCGATGGTATAAATAAAATCATGTGGATGCA**^**TTTTGCGCCGAGTGGACA---------------------------------------------------------------------------------------------------

--------------------------CCGCTGGCCCTGGACGGAAGCTAATGGACGGGCGCTGCCCCTGACTCGGCTATCGGGTAGCATTAGCAAAAGGAGATTTTCGACCCGCACACTGAAGCCGCCCGATGGTATAAATAAAATCATGTGGATGCA**^**TTTTGCGCCGAGTGGACA---------------------------------------------------------------------------------------------------

-----------------------------CTGGCCCTGGACGGAAGCTAATGGACGGGCGCTGCCCCTGACTCGGCTATCGGGTAGCATGAGCAAAAGGAGATTTTCGACCCGCACACTGAAGCCGCCCGATGGTATAAATAAAATCATGTGGATGCA**^**TTTTGCGCCGAGTGGACACTA------------------------------------------------------------------------------------------------

-----------------------------CTGGCCCTGGACGGAAGCTAATGGACGGGCGCTGCCCCTGACTCGGCTATCGGGTAGCATTAGCAAAAGGAGATTTTCGACCCGCACACTGAAGCCGCCCGATGGTATAAATAAAATCATGTGGATGCA**^**TTTTGCGCCGAGTGGACACTA------------------------------------------------------------------------------------------------

---------------------------------CCCTGGACGGAAGCTAATGGACGGGCGCTGCCCCTGACTCGGCTATCGGGTAGCATTAGCAAAAGGAGATTTTCGACCCGCACACTGAAGCCGCCCGATGGTATAAATAAAATCATGTGGATGCA**^**TTTTGCGCCGAGTGGACACTAGAGT--------------------------------------------------------------------------------------------

------------------------------------TGGACGGAAGCTAATGGACGGGCGCTGCCCCTGACTCGGCTATCGGGTAGCATTAGCAAAAGGAGATTTTCGACCCGCACACTGAAGCCGCCCGATGGTATAAATAAAATCATGTGGATGCA**^**TTTTGCGCCGAGTGGACACTAGAGTAGG-----------------------------------------------------------------------------------------

-------------------------------------GGACGGAAGCTAATGGACGGGCGCTGCCCCTGACTCGGCTATCGGGTAGCATTAGCAAAAGGAGATTTTCGAACCGCACACTGAAGCCGCCCGATGGTATAAATAAAATCATGTGGATGCA**^**TTTTGCGCCGAGTGGACACTAGAGTAGGG----------------------------------------------------------------------------------------

-------------------------------------------AAGCTAATGGACGGGCGCTGCCCCTGACTCGGCTATCGGGTAGCATTAGCAAAAGGAGATTTTCGACCCGCACACTGAAGCCGCCCGATGGTATAAATAAAATCATGTGGATGCA**^**TTTTGCGCCGCGTGGACACTAGAGTAGGGGGACAA----------------------------------------------------------------------------------

----------------------------------------------CTAATGGACGGGCGCTGCCCCTGACTCGGCTATCGGGTAGCATTAGCAAAAGGAGATTTTCGACCCGCACACTGAAGCCGCCCGATGGTATAAATAAAATCATGTGGATGCA**^**TTTTGCGCCGAGTGGACACTAGAGTAGGGGGACAAATC-------------------------------------------------------------------------------

-----------------------------------------------TAATGGACGGGCGCTGCCCCTGACTCGGCTATCGGGTAGCATTAGCAAAAGGAGATTTTCGACCCGCACACTGAAGCCGCCCGATGGTATAAATAAAATCATGTGGATGCA**^**TTTTGCGCCGAGTGGACACTAGAGTAGGGGGACAAATCG------------------------------------------------------------------------------

------------------------------------------------AATGGACGGGCGCTGCCCCTGACTCGGATATCGGGTAGCATTAGCAAAAGGAGATTTTCGACCCGCACACTGAAGCCGCCCGATGGTATAAATAAAATCATGTGGATGCA**^**TTTTGCGCCGAGTGGACACTAGAGTAGGGGGACAAATCGG-----------------------------------------------------------------------------

----------------------------------------------------GACGGGCGCTGCCCCTGACTCGGCTATCGGGTAGCATTAGCAAAAGGAGATTTTCGACCCGCACACTGAAGCCGCCCGATGGTATAAATAAAATCATGTGGATGCA**^**TTTTGCGACGAGTGGACACTAGAGTAGGGGCACAAATCGGTACC-------------------------------------------------------------------------

-------------------------------------------------------------------------------------------------------------CCCGCACACTGAAGCCGCCCGATGGTATAAATAAAATCATGTGGATGCA**^**TTTTGCGCCGAGTGGACACTAGAGTAGGGGGACAAATCGGTACCCATTCCAGCAAAAAGTCGAAGAATACTCTGCTGCAGCCCGCAAGAAGAGAGGACCAC----------------

**ACCTACATCGATAATATCCCGAACGACCGCTGGCCCTGGACGGAAGCTAATGGACGGGCGCTGCCCCTGACTCGGCTATCGGGTAGCATTAGCAAAAGGAGATTTTCGACCCGCACACTGAAGCCGCCCGATGGTATAAATAAAATCATGTGGATGCA^TTTTGCGCCGAGTGGACACTAGAGTAGGGGGACAAATCGGTACCCATTCCAGCAAAAAGTCGAAGAATACTCTGCTGCAGCCCGCAAGAAGAGAGGACCACATGAT-----------**

**S12 Data. Junction-spanning read alignments from double-insertion event.**

Multiple sequence alignment of chimeric reads extracted from four distinct junction pileups in a double-insertion event. The construct-to-genome transitions are marked with ^. Reads spanning up to each junction confirm the construct sequence, while progressively truncated reads (soft-clipped portions colored to match IGV appearance) extend into their respective flanking genomic DNA, defining precise junction boundaries at four distinct and consistent coordinates. Consensus sequences of the unaligned portions yield the flanking genomic sequences (shown in bold at bottom of each alignment). Mapping these consensus flanking sequences to a host genome reference enables disambiguation of which flanks belong to which insert and supports independent chromosomal localization of both insertions (see S12 Data).

**Left Border “Junction L1” at base 263 of construct**

--------------GACTGCTATACATAAAACCACGTTGGTGATAGACCGTTTTGGCAGTCCACCTTTTGTTCACGAAGTAGCTATGGTAAGCTACTGCGGTGTATTATCCCAAAGCT^CATAGAATGCCCTTTATAATATGAGAGGCCGTATCTCGGTATCTGA----------------------------------------------------------------------------------------------------

----------------CTGCTATACATAAAACCACGTTGGTGATAGACCGTTTTGGCAGTCCACCTTTTGTTCACGAAGTAGCTATGGTAAGCCACTGCGGTGTATTATCCCAAAGCT^CATAGAATGCCCTTTTTAATATGAGAGGCCGTATCTCGGTATCTGAAT--------------------------------------------------------------------------------------------------

-------CAAGGCCGACTGCTATACATAAAACCACGTTGGTGATAGACCGTTTTGGCAGTCCACCTTTTGTTCACGAAGTAGCTATGGTAAGCTACTGCGGTGTATTATCCCAAAGCT^CATAGAATGCCCTTTATAATATGAGAGGCCGTATCTCGG-----------------------------------------------------------------------------------------------------------

---------AGGCCGACTGCTATACATAAAACCACGTTGGTGATAGACCGTTTTGGCAGTCCACCTTTTGTTCACGAAGTAGCTATGGTAAGCTACTGCGGTGTATTATCCCAAAGCT^CATAGAATGCCCTTTATAATATGAGAGGCCGTATCTCGGTA---------------------------------------------------------------------------------------------------------

TCAGCGTCAAGGCCGACTGCTATACATAAAACCACGTTGGTGATAGACCGTTTTGGCAGTCCACCTTTTGTTCACGAAGTAGCTATGGTAAGCTACTGCGGTGTATTATCCCAAAGCT^CATAGAATGCCCTTTATAATATGAGAGGCCGT------------------------------------------------------------------------------------------------------------------

--------------GACTGCTATACATAAAACCACGTTGGTGATAGACCGTTTTGGCAGTCCACCTTTTGTTCACGAAGTAGCTATGGTAAGCTACTGCGGTGTATTATCCCAAAGCT^CATAGAATGCCCTTTATAATATGAGAGGCCGTATCTCGGTATCTGA----------------------------------------------------------------------------------------------------

--------AAGGCCGACTGCTATACATAAAACCACGTTGGTGATAGACCGTTTTGGCAGTCCACCTTTTGTTCACGAAGTAGCTATGGTAAGCTACTGCGGTGTATTATCCCAAAGCT^CATAGAATGCCCTTTATAATATGAGAGGCCGTATCTCGGT----------------------------------------------------------------------------------------------------------

---GCGTCAAGGCCGACTGCTATACATAAAACCACGTTGGTGATAGACCGTTTTGGCAGTCCACCTTTTGTTCACGAAGTAGCTATGGTAAGCTACTGCGGTGTATTATCCCAAAGCT^CATAGAATGCCCTTTATAATATGAGAGGCCGTATC---------------------------------------------------------------------------------------------------------------

TCAGCGTCAAGGCCGACTGCTATACATAAAACCACGTTGGTGATAGACCGTTTTGGCAGTCCACCTTTTGTTCACGAAGTAGCTATGGTAAGCTACTGCCGTGTATTATCCCAAAGCT^CATAGAATGCCCTTTATAATATGAGAGGCCGT------------------------------------------------------------------------------------------------------------------

--------------------TATACATAAAAGCACGTTGGTGATAGACCGTTTTGGCAGTCCACCTTTTGTTCACGAAGTAGCTATGGTAAGCTACTGCGTTGTATTATCCCAAAGCT^CATAGAATGCCCTTTATAATATGAGAGGCCGTATCTCGGTATCTGAATCATC----------------------------------------------------------------------------------------------

-----------------------------------------------------------TCCACCTTTTGTTCACGAAGTAGCTATGGTAAGCTACTGCGGTGTATTATCCCAAAGCT^CATAGAATGCCCTTTATAATATGAGAGGCCGTATCTCGGTATCTGAATCCTCTCGCTCATTGACCTGTTATAACCATGGAACTCTGGCGAG-------------------------------------------------------

--------------------------------------------------------------ACCTTTTGTTCACGAAGTAGCTATGGTAAGCTACTGCGGTGTATTATCCCAAAGCT^CATAGAATGCCCTTTATAATATGAGAGGCCGTATCTCGGTATCTGAATCCTCTCGCTCATTGACCTGTTATAACCATGGAACTCTGGCGAGTCG----------------------------------------------------

---------------------ATACATAAAACCACGTTGGTGATAGACCGTTTTGGCAGTCCACCTTTTGTTCACGAAGTAGCTATGGTAAGCTACTGCGGTGTATTATCCCAAAGCT^CATAGAATGCCCTTTATAATATGAGAGGCCGTATCTCGGTATCTGAATCCTCT---------------------------------------------------------------------------------------------

---------------------------------------------GACCGTTGTGGCAGTCCACCTTTTGTTCACGAAGTAGCTATGGTAAGCTACTGCGGTGTATTATCCCAAAGCT^CATAGAATGCCCTTTATAATATGAGAGGCCGTATCTCGGTATCTGAATCCTCTCGCTCATTGACCTGTTATAACCAT---------------------------------------------------------------------

----------------------------------------------------TTGGCAGTCCACCTTTTGTTCACGAAGTAGCTATGGTAAGCTACTGCGGTGTATTATCCCAAAGCT^CATAGAATGCCCTTTCTAATATGAGAGGCCGTATCTCGGTATCTGAATCCTCTCGCTCATTGACCTGTTATAACCATGGAACTC--------------------------------------------------------------

---------------------------------ACGTTGGTGATAGACCGTTTTGGCAGTCCACCTTTTGTTCACGAAGTAGCTATGGTAAGCTACTGCGGTGTATTATCCCAAAGCT^CATAGAATGCCCTTTATAATATGAGAGGCCGTATCTCGGTATCTGAATCCTCTCGCTCATTGACC---------------------------------------------------------------------------------

----------------------------------------------------------------CTTTTGTTCACGAAGTAGCTATGGTAAGCTACTGCGGTGTATTATCCCAAAGCT^CATAGAATGCCCTTTATAATATGAGAGGCCGTATCTCGGTATCTGAATCCTCTCGCTCATTGACCTGTTATAACCATGGAACTCTGGCGAGTCGGG--------------------------------------------------

-------------------CTATACATAAAACCACGTTGGTGATAGACCGTTTTGGCAGTCCACCTTTTGTTCACGAAGTAGCTATGGTAAGCTACTGCGGTGTATTATCCCAAAGCT^CATAGAATGCCCTTTATAATATGAGAGGCCGTATCTCGGTATCTGAATCCT-----------------------------------------------------------------------------------------------

-------------------------------------------------GTTTTGGCAGTCCACCTTTTGTTCACGAAGTAGCTATGGTAAGCTACTGCGGTGTATTATCCCAAAGCT^CATAGAATGCCCTTTATAATATGAGAGGCCGTATCTCGGTATCTGAATCCTCTCGCTCATTGACCTGTTATAACCATGGAA-----------------------------------------------------------------

------------------------------------------------------------CCACCTTTTGTTCACGAAGTAGCGATGGTAAGCTACTGCGGTGTATTATCCCAAAGCT^CATAGAATGCCCTTTATAATATGAGAGGCCGTATCTCGGTATCTGAATCCTCTCGCTCATTGACCTGTTATAACCATGGAACTCTGGCGATT------------------------------------------------------

--------------------------------------------------TTTTGGCAGTCCACCTTTTGTTCACGAAGTAGCTATGGTAAGCTACTGCGGTGTATTATCCCAAAGCT^CATAGAATGCCCTTTATAATATGAGAGGCCGTATCTCGGTATCTGAATCCTCTCGCTCATTGACCTGTTATAACCATGGAAC----------------------------------------------------------------

--------------------------------------------------------CAGTCCACCTTTTGTTCACGAAGTAGCTATGGTAAGCTACTGCGGTGTATTATCCCAAAGCT^CATAGAATGCCCTTTATAATATGAGAGGCCGTATCTCGGTATCTGAATCCTCTCGCTCATTGACCTGTTATAACCATGGAACTCTGGC----------------------------------------------------------

-------------------CTATACATAAAACCACGTTGGTGATAGACCGTTTTGGCAGTCCACCTTTTGTTCACGAAGTAGCTATGGTAAGCTACTGCGGTGTATTATCCCAAAGCT^CATAGAATGCCCTTTATAATATGGGAGGCCGTATCTCGGTATCTGAATCCT-----------------------------------------------------------------------------------------------

---------------------------------------------------------AGTCCACCTTTTGTTCACGAAGTAGCTATGGTAAGCTACTGCGGTGTATTATCCCAAAGCT^CATAGAATGCCCTTTATAATATGAGAGGCCGTATCTCGGTATCTGAATCCTCTCGCTCATTGACCTGTTATAACCATGGAACTCTGGCG---------------------------------------------------------

---------------------------------------------------------AGTCCACCTTTTGTTCACGAAGTAGCTATGGGAAGCTACTGCGGTGTATTATCCCAAAGCT^CATAGAATGCCCTTTATAATATGAGAGGCCGTATCTCGGTATCTGAATCCTCTCGCTGATTGACCTGTTATAACCATGGAACTCTGGCG---------------------------------------------------------

-------------------------------------------------------------------TTGTTCACGAAGTAGCTATGGTAAGCTACTGCGGTGTATTATCCCAAAGCT^CATAGAATGCCCTTTATAATATGAGAGGCCGTATCTCGGTATCTGAATCCTCTCGCTCATTGACCTGTTATAACCATGGAACTCTGGCGAGTCGGGGCG-----------------------------------------------

-----------------------------------GTTGGTGATAGACCGTTTTGGCAGTCCACCTTTTGTTCACGAAGTAGCTATGGTAAGCTACTGCGGTGTATTATCCCAAAGCT^CATAGAATGCCCTTTATAATATGAGAGGCCGTATCTCGGTATCTGAATCCTCTCGCTCATTGACCTG-------------------------------------------------------------------------------

-------------------------------------TGGTGATAGACCGTTTTGGCAGTCCACCTTTTGTTCACGAAGTAGCTATGGTAAGCTACTGCGGTGTATTATCCCAAAGCT^CATAGAATGCCCTTTATAATATGAGAGGCCGTATCTCGGTATCTGAATCCTCTCGCTCATTGACCTGTT-----------------------------------------------------------------------------

-------------------------ATAAAACCACGTTGGTGATAGACCGTTTTGGCAGTCCACCTTTTGTTCACGAAGTAGCTATGGTAAGCTACTGCGGTGTATTATCCCAAAGCT^CATAGAATGCCCTTTATAATATGAGAGGCCGTATCTCGGTATCTGAATCCTCTCGCT-----------------------------------------------------------------------------------------

----------------------TACATAAAACCACGTTGGTGATAGACCGTTTTGGCAGTCCACCTTTTGTTCACGAAGTAGCTATGGTAAGCTACTGCGGTGTATTATCCCAAAGCT^CATAGAATGCCCTTTATAATATGAGAGGCCGTATCTCGGTATCTGAATCCTCTC--------------------------------------------------------------------------------------------

--------------------------------------------------------------------------------AGCTATGGTAAGCTACTGCGGTGTATTATCCCAAAGCT^CATAGAATGCCCTTTATAATATGAGAGGCCGTATCTCGGTATCTGAATCCTCTCGCTCATTGACCTGTTATAACCATGGAACTCTGGCGAGTCGGGGCGAATATGGACTATA----------------------------------

------------------------------------------------------------------------------------------------------------------AGCT^CATAGAATGCCCTTTATAATATGAGAGGCCGTATCTCGGTATCTGAATCCTCTCGCTCATTGACCTGTTATAACCATGGAACTCTGGCGAGTCGGGGCGAATATGGACTATATTTAGCTCTCATGCGTGTTATATGTATTGCATCA

------------------------------------------------------------------------------------------------------------------AGCT^CATAGAATGCCCTTTATAATATGAGAGGCCGTATCTCGGTATCTGAATCCTCTCGCTCATTGACCTGTTATAACCATGGAACTATGGCGAGTCGGGGCGAATATGGACTATATTTAGCTCTCATGCGTGTTATATGTATTGCATCA

----------------------------------------------------------------------------------------------ACTGCGCTGTATTATCCCAAAGCT^CATAGAATGCCCTTTATAATATGAGAGGCCGTATCTCGGTATCTGAATCCTCTCGCTCATTGACCTGTTATAACCATGGAACTCTGGCGAGTCGGGGCGAATATGGACTATATTTAGCTCTCATGC--------------------

-------------------------------------------------------------------------------TAGCTATGGTAAGCTACTGCGGTGTATTATCCCAAAGCT^CATAGAATGCCCTTTATAATATGAGAGGCCGTATCTGGGTATCTGAATCCTCTCGCTCATTGACCTGTTATAACCATGGAACTCTGGCGAGTCGGGGCGAATATGGACTAT-----------------------------------

--------------------------------------------------------------------------CGAAGTAGCTATGGTAAGCTACTGCGGTGTATTATCCCAAAGCT^CATAGAATGCCCTTTATAATATGAGAGGCCGTATCTCGGTATCTGAATCCTCTCGCTCATTGACCTGTTATAACCATGGAACTCTGGCGAGTCGGGGCGAATATGG----------------------------------------

-----------------------------------------------------------------------------------TATGGTAAGCTACTGCGGTGTATTATCCCAAATCT^CATAGAATGCCCTTTATAATATGAGAGGCCGTATCTCGGTATCTGAATCCTCTCGCTCATTGACCTGTTATAACCATGGAACTCTGGCGAGTCGGGGCGAATATGGACTATATTT-------------------------------

---------------------------------------------------------------------------------------------------------------CAAAGCT^CATAGAATGCCCTTTATAATATGAGAGGCCGTATCTCGGTATCTGAATCCTCTCGCTCATTGACCTGTTATAACCATGGAACTCTAGCGAGTCGGGGCGAATATGGACTATATTTAGCTCTCATGCGTGTTATATGTATTGCA---

----------------------------------------------------------------------------------------TAAGCTACTGCGGTGTATTATCCCAAAGCT^CATAGAATGCCCTTTATAATATGAGAGGCCGTATCTCGGTATCTGAATCCTCTCGCTCATTGACCTGTTATAACCATGGAACTCTGGCGAGTCGGGGCGAATATGGACTATATTTAGCTC--------------------------

---------------------------------------------------------------------------------------GTAAGCTACTGCGGTGTATTATCCCAAAGCT^CATAGAATGCCCTTTATAATATGAGAGGCCGTATCTCGGTATCTGAATCCTCTCGCTCATTGACCTGTTATAACCATGGAACTCTGGCGAGTCGGGGCGAATATGGACTATATTTAGCT---------------------------

----------------------------------------------------------------------------------------------------------------AAAGCT^CATAGAATGCCCTTTATAATATGAGAGGCCGTATCTCGGTATCTGAATCCTCTCGCTCATTGACCTGTTATAACCATGGAACTCTGGCGAGTCGGGGCGAATATGGACTATATTTAGCTCTCATGCGTGTTATATGTATTGCAT--

-------------------------------------------------------------------------------------------GCTACTGCGGTGTATTATCCCAAAGCT^CATAGAATGCCCTTTATAATATGAGAGGCCGTATCTCGGTATCTGAATCCTCTCGCTCATTGACCTGTTATAACCATGGAACTCTGGCGAGTCGGGGCGAATATGGACTATATTTAGCTCTCA-----------------------

--------------------------------------------------------------------TGTTCACGAAGTAGCTATGGTAAGCTACTGCGGTGTATTATCCCAAAGCT^CATAGAATGCCCTTTATAATATGAGAGGCCGTATCTCGGTATCTGAATCCTCTCGCTCATTGACCTGTTATAACCATGGAACTCTGGCGAGTCGGGGCGA----------------------------------------------

**TCAGCGTCAAGGCCGACTGCTATACATAAAACCACGTTGGTGATAGACCGTTTTGGCAGTCCACCTTTTGTTCACGAAGTAGCTATGGTAAGCTACTGCGGTGTATTATCCCAAAGCT^CATAGAATGCCCTTTATAATATGAGAGGCCGTATCTCGGTATCTGAATCCTCTCGCTCATTGACCTGTTATAACCATGGAACTCTGGCGAGTCGGGGCGAATATGGACTATATTTAGCTCTCATGCGTGTTATATGTATTGCATCA**

>L1_Flank_Consensus

TCAGCGTCAAGGCCGACTGCTATACATAAAACCACGTTGGTGATAGACCGTTTTGGCAGTCCACCTTTTGTTCACGAAGTAGCTATGGTAAGCTACTGCGGTGTATTATCCCAAAGCT

**Left Border “Junction L2”at base 280 of construct**

----ATACCATCCTTCGATTCGTGGCTGTAGATAGATCTTTAATAGTTCACTTATACCTTGGATTTAACGGCCGATTCTAAAACACTTCGAGGGCTAGTCAAGGAGGCCATACTAACC^AATATGAGAGGCCGTCTCTCGGTATCTGAATCCTCT----------------------------------------------------------------------------------------------------------------

----------------GATTCGTGGCTGTAGATAGATCTTAAATAGTTCACTTATACCTTGGATTTAACGGCCGATTCTAAAACACTTCGAGGGCTAGTCAAGGCGGCCATACTAACC^AATATGAGAGGCCGTATCTCGGTATCTGAATCCTCTCGCTCATTGACC----------------------------------------------------------------------------------------------------

-----TACCATCCTTCGATTCGTGGCTGTAGATAGATCTTAAATAGTTCACTTATACCTTGGATTTAACGGCCGATTCTAAAACACTTCGAGGGCTAGTCAAGGAGGCCATACTAACC^AATATGAGAGGCCGTATCTCGGTATCTGAATCCTCTC---------------------------------------------------------------------------------------------------------------

-----------------ATTCGTGGCTGTAGATAGATCTTAAATAGTTCACTTATACCTTGGATTTAACGGCCGATTCTAAAACACTTCGAGGGCTAGTCAAGGAGGCCATACTAACC^AATATGAGAGGCCGTATCTCGGTATCTGAATCCTCTCGCTCATTGACCT---------------------------------------------------------------------------------------------------

---GATACCATCCTTCGATTCGTGGCTGTAGATAGATCTTAAATAGTTCACTTATACCCTGGATTTAACGGCCGATTCTAAAACACTTCGAGGGCTAGTCAAGGAGGCCATACTAACC^AATATGAGAGGCCGTATCTCGGTATCTGAATCCTC-----------------------------------------------------------------------------------------------------------------

--------CATCCTTCGATTCGTGGCTGTAGATAGATCTTAAATAGTTCACTTATACCTTGGATTTAACGGCCGATTCTAAAACACTTCGAGGGCTAGTCAAGGAGGCCATACTAACC^AATATGAGAGGCCGTATCTCGGTATCTGAATCCTCTCGCT------------------------------------------------------------------------------------------------------------

AAAGATACCATCCTTCGATTCGTGGCTGTAGATAGATCTTAAATAGTTCACTTATACCTTGGATTTAACGGCCGATTCTAAAACACTTCGAGGGCTAGTCAAGGAGGCCATACTAACC^AATATGAGAGGCCGTATCTCGGTATCTGAATC--------------------------------------------------------------------------------------------------------------------

--------CATCCTTCTATTCGTGGCTGTAGATAGATCTTAAATAGTTCACTTATACCTTGGATTTAACGGCCGATTCTAAAACACTTCGAGGGCTAGTCAAGGAGGCCATACTAACC^AATATGAGAGGCCGTATCTCGGTATCTGAATCCTCTCGCT------------------------------------------------------------------------------------------------------------

-----------------ATTCGTGGCTGTAGATAGATCTTAAATAGTTCACTTATACCTTGGATTTAACGGCCGATTCTAAAACACTTCGAGGGCTAGTCAAGGAGGCCATACTAACC^AATATGAGAGGCCGTATCTCGGTATCTGAATCCTCTCGCTCATTGACCT---------------------------------------------------------------------------------------------------

----ATACCACCCTTCGATTCGTGGCTGTAGATAGATCTTAAATAGTTCACTTATACCTTGGATTTAACGGCCGATTCTAAAACACTTCGAGGGCTAGTCAAGGAGGCCATACTAACC^AATATGAGAGGCCGTATCTCGGTATCTGAATCCTCT----------------------------------------------------------------------------------------------------------------

---------------------------------------------------------CTTGGATTTAACGGCCGATTCTAAAACACTTCGAGGGCTAGTCAAGGAGGCCATACTAACC^AATATGAGAGGCCGTATCTCGGTATCTGAATCCTCTCGCTCATTGACCTGTTATAACCATGGAACTCTGGCGAGTCGGGGCGAATATGG-----------------------------------------------------------

-----------------------------------------AATAGTTCACTTATACCTTGGATTTAACGGCCGATTCTAAAACACTTCGAGGGCTAGTCAAGGAGGCCATACTAACC^AATATGAGAGGCCGTATCTCGGTATCTGAATCCTCTCGCTCATTGACCTGTTATAACCATGGAACTCTGGCGA---------------------------------------------------------------------------

------------------------------GATAGATCTTAAATAGTTCACTTATACCTTGGATTTAACGGCCGATTCTAAAACACTTCGAGGGCTAGTCAAGGAGGCCATACTAACC^AATATGAGAGGCCGTATCTCGGTATCTGAATCCTCTCGCTCATTGACCTGTTATAACCATGG--------------------------------------------------------------------------------------

------------------------------------------------------TACCTTGGATTTAACGGCCGATTCTAAAACACTTCGAGGGCTAGTCAAGGAGGCCATACTAACC^AATATGAGAGGCCGTATCTCGGTATCTGAATCCTCTCGCTCATTGACCTGTTATAACCATGGAACTCTGGCGAGTCGGGGCGAATA--------------------------------------------------------------

--------------------------CGTAGATAGATCTTAAATAGTTCACTTATACCTTGGATTTAACGGCCGATTCTAAAACACTTCGAGGGATAGTCAAGGAGGCCATACTAACC^AATATGAGAGGCCGTATCTCGGTATCTGAATCCTCTCGCTCATTGACCTGTTATAACC------------------------------------------------------------------------------------------

----------------------TGGCTGTAGATAGATCTTAAATAGTTCACTTATACCTTGGATTTAACGGCCGATTCTAAAACAGTTCGAGGGCTAGTCAAGGAGGCCATACTAACC^AATATGAGAGGCCGTATCTCGGTATCTGAATCCTCTCGCTCATTGACCTGTTAT----------------------------------------------------------------------------------------------

-------------------------------------CTTAAATAGTTCACTTATACCTTGGATTTAACGGCCGATTCTAAAACACTTCGAGGGCTAGTCAAGGAGGCCATACTAACC^AATATGAGAGGCCGTATCTCGGTATCTGAATCCTCTCGCTCATTGACCTGTTATAACCATGGAACTCTG-------------------------------------------------------------------------------

----------------------------------------------------------TTGGATTTAACGGCCGATTCTAAAACACTTCGAGGGCTAGTCAAGGAGGCCATACTAACC^AATATGAGAGGCCGTATCTCGGTATCTGAATCCTCTCGCTCATTGACCTGTTATAACCATGGAACTCTGGCGAGTCGGGGCGAATATGGA----------------------------------------------------------

-------------------------------------CTTAAATAGTTCACTTATACCTTGGATTTAACGGCCGATTCTAAAACATTTCGAGGGCTAGTCAAGGAGGCCATACTAACC^AATATGAGAGGCCGTATCTCGGTATCTGAATCCTCTCGCTCATTGACCTGTTATAACCATGGAACTCTG-------------------------------------------------------------------------------

---------------------GTGGCTGTAGATAGATCTTAAATAGTTCACTTATACCTTGGATTTAACGGCCGATTCTAAAACACTTCGAGGGCTAGTCAAGGAGGCCATACTAACC^AATATGAGAGGCCGTATCGCGGTATCTGAATCCTCTCGCTCATTGACCTGTTA-----------------------------------------------------------------------------------------------

--------------------------------------------AGTTCACTTATACCTTGGATTTAACGGCCGATTCTAAAACACTTCGAGGGCTAGTCAAGGAGGCCATACTAACC^AATATGAGAGGCCGTATCTCGGTATCTGAATCCTCTCGCTCATTGACCTGTTATAACCATGGAACTCTGGCGAGTC------------------------------------------------------------------------

----------------------------------------AAATAGTTCACTTATACCTTGGATTTAACGGCCGATTCTAAAACACTTCGAGGGCTAGTCAAGGAGGCCATACTAACC^AATATGAGAGGCCGTATCTCGGTATCTGAATCCTCTCGCCCATTGACCTGTTATAACCATGGAACTCTGGCG----------------------------------------------------------------------------

-------------------------------------------------ACTTATACCTTGGATTTAACGGCCGATTCTAAAACACTTCGAGGGCTAGTCAAGGAGGCCATACTAACC^AATATGAGAGGCCGTATCTCGGTATCTGAATCCTCTCGCTCATTGACCTGTTATAACCATGGAACTCTGGCGAGTCGGGGC-------------------------------------------------------------------

----------------------------------------------------------TTGGATTTAACGGCCGATTCTAAAACACTTCGAGGGCTAGTCAAGGAGGCCATACTAACC^AATATGAGAGGCCGTATCTCGGTATCTGAATCCTCTCGCTCATTGACCTGTTATAACCATGGAACTCTGGCGAGTCGGGGCGAATATGGA----------------------------------------------------------

--------------------------------TAGATCTTAAATAGTTCACTTATACCTTGGATTTAACGGCCGATTCTAAAACACTTCGAGGGCTAGTCAAGGAGGCCATACTAACC^AATATGAGAGGCCGTATCTCGGTATCTGAATCCTCTCGCTCATTGACCTGTTATAACCATGGAA------------------------------------------------------------------------------------

--------------------------------------------------CTTATACCTTGGATTTAACGGCCGATTCTAAAACACTTCGAGGGCTAGTCAAGGAGGCCATACTAACC^AATATGAGAGGCCGTATCTCGGTATCTGAATCCTCTCGCTCATTGACCTGTTATAACCATGGAACTCTGGCGAGTCGGGGCG------------------------------------------------------------------

---------------------------------------------------------CTTGGATTTAACGGCCGATTCTAAGACACTTCGAGGGCTAGTCAAGGAGGCCATACTAACC^AATATGAGAGGCCGTATCTCGGTATCTGAATCCTCTCGCTCATTGACCTGTTATAACCATGGAACTCTGGCGAGTCGGGGCGAATATGG-----------------------------------------------------------

--------------------------------------------------------------------------------------------------------------TACTAACC^AATATGAGAGGCCGTATCTCGGTATCTGAATCCTCTCGCTCATTGACCTGTTATAACCATGGAACTCTGGCGAGTCGGGGCGAATATGGACTATATTTAGCTCTCATGCGTGTTATATGTATTGCATCATGTCAAATTTACA------

------------------------------------------------------------------------------TAAAACACTTCGAGGGCTAGTCAAGGAGGCCATACTAACC^AATATGAGAGGCCGTATCTCGGTATCTGAATCCTCTCGCTCATTGACCTGTTATAACCATGGAACTCTGGCGAGTCGGGGCGAATATGGACTATATTTAGCTCTCAGGCG--------------------------------------

-------------------------------------------------------------------------------------------------GTCAAGGAGGCCATACAAACC^AATATGAGAGGCCGTATCTCGGTATCTGAATCCTCTCGCTCATTGACCTGTTATAACCATGGAACTCTGGCGAGTCGGGGCGAATATGGACTATATTTAGCTCTCATGCGTGTTATATGTATTGCACCA-------------------

--------------------------------------------------------------------------------------------------------AGGCCATACTAACC^AATATGAGAGGCCGTATCTCGGTATCTGAATCCTCTCGCTCATTGACCTGTTATAACCATGGAACTCTGGCGAGTCGGGGCGAATATGGACTATATTTAGCTCTCATGCGTGTTATATGTATTGCATCATGTCAAA------------

------------------------------------------------------------------------CGATTCTAAAACACTTCGAGGGCTAGTCAAGGAGGCCATACTAACC^AATATGAGAGGCCGTATCTCGGTATCTGAATCCTCTCGCTCATTTACCTGTTATAACCATGGAACTCTGGCGAGTCGGGGCGAATATGGACTATATTTAGCTCT--------------------------------------------

-----------------------------------------------------------------------------CTAAAACACTTCGAGGGCTAGTCAAGGAGGCCATACTAACC^AATATGAGAGGCCGTATCTCGGTATCTGAATCCTCTCGCTCATTGACCTGTTATAACCATGGAACTCTGGCGAGTCGGGGCGAATATGGACTATATTTAGCTCTCATGC---------------------------------------

--------------------------------------------------------------------------------------------------------------------CC^AATATGAGAGGCCGTATCTCGGTATCTGAATCCTCTCGCTCATTGACCTGTTATAACCATGGAACTCTGGCGAGTCGGGGCGAATATGGACTATATTTAGCTCTCATGCGTGTTATACGTATTGCATCATGTCAAATTTACACCAGTT

-------------------------------------------------------------------------GATTCTAAAACACTTCGAGGGCTAGTCAAGGAGGCCATACTAACC^AATATGAGAGGCCGTATCTCGGTATCTGAATCCTCTCGCTCATTGACCTGTTATAACCATGGAACTCTGGCGAGTCGGGGCGAATATGGACTATATTTAGCTCTC-------------------------------------------

--------------------------------------------------------------------------------------------------------------------CC^AATATGAGAGGCCGTATCTCGGTATCTGAATCCTCTCGCTCATTGACCTGTTATAACCATGGAACTCTGGCGAGTCGGGGCGAATATGGACTATATTTAGCTCTCATGCGTGTTATATGTATTGCATCATGTCAAATTTACACCAGTT

------------------------------------------------------------------------------------ACTTCGAGGGCTAGTCAAGGAGGCCATACTAACC^AATATGAGAGGCCGTATCTCGGTATCTGAATCCTCTCGCTCATTGACCTGTTATAACCATGGAACTCTGGCGAGTCGGGGCGAATATGGACTATATTTAGCTCTCATGCGTGTTAT--------------------------------

-----------------------------------------------------------------------------------------------------AGGAGGCCATACTAACC^AATATGAGAGGCCGTATCTCGGTATCTGAATCCTCTCGCTCATTGACCTGTTATAACCATGGAACTCTGGCGAGTCGGGGCGAATATGGACTATATTTAGCTCTCATGCGTGTTATATGTATTGCATCAAGTC---------------

-------------------------------------------------------------------------------AAAACACTTCGAGGGCTAGTCAAGGAGGCCATACTAACC^AATATGAGAGGCCGTATCTCGGTATCTGAATCCTCTCGCTCATTGACCTGTTATAACCATGGAACTCTGGCGAGTCGGGGCGAATATGGACTATATTTAGCTCTCATGCGT-------------------------------------

--------------------------------------------------------------------------------------------------------------TACTAACC^AATATGAGAGGCCGTATCTCGGTATCTGAATCCTCTCGCTCATTGACCTGTTATAACCATGGAACTCTGGCGAGTCGGGGCGAATATGGACTATATTTAGCTCTCATGCGTGTTATATGTATTGCATCATGTCAAATTTACA------

--------------------------------------------------------------------------------------------GGCTAGTCAAGGAGGCCATACTAACC^AATATGAGAGGCCGTATCTCGGTATCTGAATCCTCTCGCTCATTGACCTGTTATAACCATGGAACTCTGGCGAGTCGGGGCGAATATGGACTATATTTAGCTCTCATGCGTGTTATATGTATTG------------------------

**---GATACCATCCTTCGATTCGTGGCTGTAGATAGATCTTAAATAGTTCACTTATACCTTGGATTTAACGGCCGATTCTAAAACACTTCGAGGGCTAGTCAAGGAGGCCATACTAACC^AATATGAGAGGCCGTATCTCGGTATCTGAATCCTCTCGCTCATTGACCTGTTATAACCATGGAACTCTGGCGAGTCGGGGCGAATATGGACTATATTTAGCTCTCATGCGTGTTATATGTATTGCATCATGTCAAATTTACACCAGTT**

>L2_Flank_Consensus

GATACCATCCTTCGATTCGTGGCTGTAGATAGATCTTAAATAGTTCACTTATACCTTGGATTTAACGGCCGATTCTAAAACACTTCGAGGGCTAGTCAAGGAGGCCATACTAACC

**Right Border “Junction R1” at base 3,194 of construct**

-------------------------------------------------------------------------------------------------GACCGCCCGAGACGGAGGCCCGTCGAAACGGTCAAAGCTGGCCC^ACCACGCGGGTGTTTATATCCCCAATGGGACAGGCTCCCACGTTCGGCTATTGACTCAGAAAATTGACAGCTGAGTGCGCATAATCATAGATAGGCACCTACAGTC--------------

--------------------------------------------------------------------------------------------------ACCGCCCGAGACGGAGGCCCGTCGAAACGGTCAAAGCTGGCCC^ACCACGCGGGTGTTTAAATCCCCAATGGGACAGGCTCCCACGTTCGGCTATTGACTCAGAAAATTGACAGCTGAGTGCGCATAATCATAGATAGGCACCTACAGTCA-------------

--------------------------------------------------------------------------------------------------ACCGCCCGAGACGGAGGCCCGTCGAAACGGTCAAAGCTGGCCC^ACCACGCGGGTGTTTATATCCCCAATGGGACAGGCTCCCACGTTCGGCTATTGACTCAGAAAATTGACAGCTGAGTGCGCATAATCATAGATAGGCACCTACAGTCA-------------

-----------------------------------------------------------------------------------------------------GCCCGAGACGGAGGCCCGTCGAAACGGTCAAAGCTGGCCC^ACCACGCGGGTGTTTATATCCCCAATGGGACAGGCTCCCACGTTCGGCTATTGACTCAGAAAATTGACAGCTGAGTGCGCATAATCATAGATAGGCACCTACAGTCAACA----------

------------------------------------------------------------------------------------------------------CCCGAGACGGAGGCCCGTCGAAACGGTCAAAGCTGGCCC^ACCACGCGGGTGTTTATATCCCCAATGGGACAGGCTCCCACGTTCGGCTATTGACTCAGAAAATTGACAGCTGAGTGCGCATAATCATAGATAGGCACCTACAGCCAACAA---------

---------------------------------------------------------------------------------------------------------------GAGGCCCGTCGAAACGGTCAAAGCTGGCCC^ACCACGCGGGTGTTTATATCCCCAATGGGACAGGCTCCCACGTTCGGCTATTGACTCAGAAAATTGACAGCTGAGTGCGCATAATCATAGATAGGCACCTACAGTCAACAACTCCATACA

--------------------------------------------------AGCGCACGGCTTATCTCAAGAAAATCGACGCGGAAGAATGTTGCCAGGACCGCCCGAGACGGAGGCCCGTCGAAACGGTCAAAGCTGGCCC^ACCACGCGGGTGTTTATATCCCCAATGGGACAGGCTCCCACGTTCGGCTATTGACTCAG-------------------------------------------------------------

---------------------------------------------------GCGCACGGCTTATCTCAAGAAAATCGACGCGGAAGAATGTTGCCAGGACCGCCCGAGACGGAGGCCCGTCGAAACGGTCAAAGCTGGCCC^ACCACGCGGGTGTTTATATCCCCAATGGGACAGGCTCCCACGTTCGGCTATTGACTCAGA------------------------------------------------------------

--------------------------------------------------------CGGCTTATCTCAAGAAAATCGACGCGGAAGAATGTTGCCAGGACCGCCCGAGACGGAGGCCCGTCGAAACGGTCAAAGCTGGCCC^ACCACGCGGGTGTTTATATCCCCAATGGGACAGGCTCCCACGTTCGGCTATTGACTCAGAAAATT-------------------------------------------------------

---------------------------------------------------------GGCTTATCTCAAGAAAATCGACGCGGAAGAATGTTGCCAGGACCGCCCGAGACGGAGGCCCGTCGAAACGGTCAAAGCTGGCCC^ACCACGCGGGTGTTTATATCCCCAATGGGACAGGCTCCCACGTTCGGCTATTGACTCAGAAAATTG------------------------------------------------------

-------------------------------------------------------------TATCTCAAGAAAATCGACGCGGAAGAATGTTGCCAGGACCGCCCGAGACGGAGGCCCGTCGAAACGGTCAAAGCTGGCCC^ACCACGCGGGTGTTTATATCCCCAATGGGACAGGCTCCCACGTTCGGCTATTGACTCAGAAAATTGACAG--------------------------------------------------

-------------------------------------------------------------TATCTCAAGAAAATCGACGCGGAAGAATGTTGCCAGGACCGCCCGAGACGGAGGCCCGTCGAAACGGTCAAAGCTGGCCC^ACCACGCGGGTGTTTATATCCCCAATGGGACAGGCTCCCACGTTCGGCTATTGACTCAGAAAATTGACAG--------------------------------------------------

-----------------------------------------------------------------------AAATCGACGCGGAAGAATGTTGCCAGGACCGCCCGAGACGGAGGCCCGTCGAAACGGTCAAAGCTGGCCC^ACCACGCGGGTGTTTATATCCCCAATGGGACAGGCTCCCACGTTCGGCTATTGACTCAGAAAATTGACAGCTGAGTGCGC----------------------------------------

--------------------------------------------------------------------------TCGACGCGGAAGAATGTTGCCAGGACCGCCCGAGACGGAGGCCCGTCGAAACGGTCAAAGCTGGCCC^ACCACGCGGGTGTTTATATCCCCAATGGGACAGGCTCCCACGTTCGGCTATTGACTCAGAAAATTGACAGCTGAGTGCGCATA-------------------------------------

--------------------------------------------------------------------------TCGAAGCGGAAGAATGTTGCCAGGACCGCCCGAGACGGAGGCCCGTCGAAACGATCAAAGCTGGCCC^ACCACGCGGGTGTTTATATCCCCAATGGGACAGGCTCCCACGTTCGGCTATTGACTCAGAAAATTGACAGCTGAGTGCGCATA-------------------------------------

-----------------------------------------------------------------------------ACGCGGAAGAATGTTACCAGGACCGCCCGAGACGGAGGCCCGTCGAAACGGTCAAAGCTGGCCC^ACCACGCGGGTGTTTATATCCCCAATGGGACAGGCTCCCACGTTCGGCTATTGACTCAGAAAATTGACAGCTGAGTGCGCATAATC----------------------------------

---------------------------------------------------------------------------------GGAAGAATGTTGCCAGGACCGCCCGAGACGGAGGCCCGTCGAAACGGTCAAAGCTGGCCC^ACCACGCGGGTGTTTATATCCCCAATGGGACAGGCTCCCACGTTCGGCTATTGACTCAGAAAATTGACAGCTGAGTGCGCATAATCATAG------------------------------

-----------------------------------------------------------------------------------AAGAATGTTGCCAGGACCGCCCGAGACGGAGGCCCGTCGAAACGGTCAAAGCTGGCCC^ACCACGCGGGTGTTTATATCCCCAATGGGACAGGCTCCCACGTTCGGCTATTGACTCAGAAAATTGACAGCTGAGTGCGCATAATCATAGAT----------------------------

------------------------------------------------------------------------------------AGAATGTTGCCAGGACCGCCCGAGACGGAGGCCCGTCGAAACGGTCAAAGCTGGCCC^ACCACGCGGGTGTTTATATCCCCAATGGGACAGGCTCCCACGTTCGGCTATTGACTCAGAAAATTGACAGCTGAGTGCGCATAATCATAGATA---------------------------

--------------------------------------------------------------------------------------AATGTTGCCAGGACCGCCCGAGACGGAGGCCCGTCGAAACGGTCAAAGCTGGCCC^ACCACGCGGGTGTTTATATCCCCAATGGGACAGGCTCCCACGTTCGGCTATTGACTCAGAAAATTGACAGCTGAGTGCGCATAATCATAGATAGG-------------------------

CATGCCCTAACCCCACGCACTCATATCCGCGTGCTAATAGTTTGCTACAAAGCGCACGGCTTATCTCAAGAAAATCGACGCGGAAGAATGTTGCCAGGACCGCCCGAGACGGAGGCCCGTCGAAACGGTCAAAGCTGGCCC^AACACGCGG---------------------------------------------------------------------------------------------------------------

-ATGCCCTAACCCCACGCACTCATATCCGCGTGCTAATAGTTTGCTACAAAGCGCACGGCTTATCTCAAGAAAATCGACGCGGAAGAATGTTGCCAGGACCGCCCGAGACGGAGGCCCGTCGAAACGGTCAAAGCTGGCCC^ACCACGCGGG--------------------------------------------------------------------------------------------------------------

--TGCCCTAACCCCACGCACTCATATCCGCGTGCTAATAGTTTGCTACAAAGCGCACGGCTTATCTCAAGAAAATCGACGCGGAAGAATGTTGCCAGGACCGCCCGAGACGGAGGCCCGTCGAAACGGTCAAAGCTGGCCC^ACCACGCGGGT-------------------------------------------------------------------------------------------------------------

--------AACCCCACGCACTCATATCCGCGTGCTAATAGTTTGCTACAAAGCGCACGGCTTATCTCAAGAAAATCGACGCGGAAGAATGTTGCCAGGACCGCCCGAGACGGAGGCCCGTCGAAACGGTCAAAGCTGGCCC^ACCACGCGGGTGTTTAT-------------------------------------------------------------------------------------------------------

-----------CCCACGCACTCATATCCGCGTGCTAATAGTTTGCTACAAAGCGCACGGCTTATCTCAAAAAAATCGACGCGGAAGAATGTTGCCAGGACCGCCCGAGACGGAGGCCCGTCGAAACGGTCAAAGCTGGCCC^ACCACGCGGGTGTTTATATC----------------------------------------------------------------------------------------------------

-------------CACGCACTCATATCCGCGTGCTAATAGTTTGCTACAAAGCGCACGGTTTATCTCAAGAAAATCGACGCGGAAGAATGTTGCCAGGACCGCCCGAGACGGAGGCCCGTCGAAACGGTCAAAGCTGGCCC^ACCACGCGGGTGTTTATATCCC--------------------------------------------------------------------------------------------------

-----------------CACTCATATCCGCGTGCTAATAGTTTGCTACAAAGCGCACGGCTTATCTCAAGAAAATCGACGCGGAAGAATGTTGCCAGGACCGCCCGAGACGGAGGCCCGTCGAAACGGTCAAAGCTGGCCC^ACCACGCGGGTGTTTATATCCCCAAT----------------------------------------------------------------------------------------------

---------------------CATATCCGCGTGCTAATAGTTTGCTACAAAGCGCACGGCTTATCTCAAGAAAATCGACGCGGAAGAATGTTGCCAGGACCGCCCGAGACGGAGGCCCGTCGAAACGGTCAAAGCTGGCCC^ACCACGCGGGTGTTTATATCCCCAATGGGA------------------------------------------------------------------------------------------

-------------------------TCCGCGTGCTAATAGTTTGCTACAAAGCGCACGGCTTATCTCAAGAAAATCGACGCGGAAGAATGTTGCCAGGACCGCCCGAGACGGAGGCCCGTCGAAACGGTCAAAGCTGGCCC^ACCACGCGGGTGTTTATATCCCCAATGGGACAGG--------------------------------------------------------------------------------------

--------------------------CCGCGTGCTAATAGTTTGCTACAAAGCGCACGGCTTATCTCAAGAAAATCGACGCGGAAGAATGTTGCCAGGACCGCCCGAGACGGAGGCCCGTCGAAACGGTCAAAGCTGGCCC^ACCACGCGGGTGTTTATATCCCCAATGGGACAGGC-------------------------------------------------------------------------------------

--------------------------------------AGTTTGCTACAAAGCGCACGGCTTATCTCAAGAAAATCGACGCGGAAGAATGTTGCCAGGACCGCCCGAGACGGAGGCCCGTCGAAACGGTCAAAGCTGGCCC^ACCACGCGGGTGTTTATATCCCCAATGGGACAGGCTCCCACGTTCGG-------------------------------------------------------------------------

--------------------------------------AGTTTGCTACAAAGCTCACGGCTTATCTCAAGAAAATCGACGCGGAAGAATGTTGCCAGGACCGCCCGAGACGGAGGCCCGTCGAAACGGTCAAAGCTGGCCC^ACCACGCGGGTGTTTATATCCCCAATGGGACAGGCTCCCACGTTCGG-------------------------------------------------------------------------

**-ATGCCCTAACCCCACGCACTCATATCCGCGTGCTAATAGTTTGCTACAAAGCGCACGGCTTATCTCAAGAAAATCGACGCGGAAGAATGTTGCCAGGACCGCCCGAGACGGAGGCCCGTCGAAACGGTCAAAGCTGGCCC^ACCACGCGGGTGTTTATATCCCCAATGGGACAGGCTCCCACGTTCGGCTATTGACTCAGAAAATTGACAGCTGAGTGCGCATAATCATAGATAGGCACCTACAGTCAACAA---------**

>R1_Flank_Consensus

ACCACGCGGGTGTTTATATCCCCAATGGGACAGGCTCCCACGTTCGGCTATTGACTCAGAAAATTGACAGCTGAGTGCGCATAATCATAGATAGGCACCTACAGTCAACAA

**Right Border “Junction R2” at base 3,416 of construct**

-----------------------------------------GGACGGGCGCTGCCCCTGACTCGGCTATCGGGTAGCATTAGCAAAAGGAGATTTTCGACCCGCACACTGAAGCCGCCCGATGGTATAAATAAAATCATGTGGATGCA^TTTTGCGCCGAGTGGACACTAGAGTAGGGGGACAAATCGGTAC-----------------------------------------------------------------------------

-----------------------------------------GGACGGGCGCTGCCCCTGACTCGGCTATCGGGTAGCATTAGCAAAAGGAGATTTTCGACCCGCACACTGAAGCCGCCCGATTGTATAAATAAAATCATGTGGATGCA^TTTTGCGCCGAGTGGACACTAGAGTAGGGGGACGAATCGGTAC-----------------------------------------------------------------------------

---------------------------------------------GGGCGCTGCCCCTGACTCGGCTATCGGTTAGCATTAGCAAAAGGAGATTTTCGACCCGCACACTGAAGCCGCCCGATGGTATAAATAAAATCATGTGGATGCA^TTTTGCGCCGAGTGGACACTAGAGTAGGGGGACAAATCGGTACCCAT-------------------------------------------------------------------------

---------------------------------------------GGGCGCTGCCCCTGACTCGGCTATCGGGTAGCATTAGCAAAAGGAGATTTTCGACCCGCACACTGAAACCGCCCGATGGTATAAATAAAATCATGTGGATGCA^TTTTGCGCCGAGTGGACACTAGAGTAGGGGGACAAATCGGTACCCAT-------------------------------------------------------------------------

----------------------------------------------GCCGCTGCCCCTGACTCGGCTATCGGGTAGCATTAGCAAAAGGAGATTTTCGACCCGCACACTGAAGCCGCCCGATGGTATAAATAAAATCATGTGGATGCA^TTTTGCGCCGAGTGGACACTAGAGTAGGGGGACAAATCGGTCCCCATT------------------------------------------------------------------------

------------------------------------------------CGCTGCCCCTGACTCGGCTATCGGGTAGCATTAGCAAAAGGAGATTTTCGACCCGCACACTGAAGCCGCCCGATGGTATAAATAAAATCATGTGGATGCA^TTTTGCGCCGAGTGGACACTAGAGTAGGGGGACAAATCGGTACCCATTCC----------------------------------------------------------------------

------------------------------------------------CGCTGCCCCTGACTCGGCTATCGGGTAGCATTAGCAAAAGGAGATTTTCGACCCGCACACTGAAGCCGCCCGATGGTATAAATAAAATCATGTGGATGCA^TTTTGCGCCGAGTGGACACTAGAGTAGGGGGACAAATCGGTACCCATTCC----------------------------------------------------------------------

-----------------------------------------------------------------------------------------------------CGCACACTGAAGCCGCCCGATGGTATAAATAAAATCATGTGGATGCA^TTTTGCGCCGAGTGGACACTAGAGTAGGGGGACAAATCGGTACCCATTCCAGCAAAAAGTCGAAGAATACTCTGCTGCAGCCCGCAAGAAGAGAGGACCACAT-----------------

--------------------------------------------------------------------------------------------------------ACACTGAAGCCGCCCGATGGTATAAATAAAATCATGTGGATGCA^TTTTGCGCCGAGTGGACACTAGAGTAGGGGGACAAATCGGTACCCATTCCAGCAAAAAGTCGAAGAATACTCTGCTGCAGCCCGCAAGAAGAGAGGACCACATGAT--------------

----------------------------------------------------------------------------------------------------------ACTGAAGCCGCCCGATGGTATAAATAAAATCATGTGGATGCA^TTTTGCGCCGAGTGGACACTAGAGTAGGGGGACAAATCGGTACCCATTCCAGCAAAAAGTCGAAGAATACTCTGCTGCAGCCCGCAAGAAGAGAGGACCACATGATTA------------

-----------------------------------------------------------------------------------------------------------CTGAAGCCGCCCGATGGTATAAATAAAATCATGTGGATGCA^TTTTTCGCCGAGTGGACACTAGAGTAGGGGGACAAATCGGTACCCATTCCAGCAAAAAGTCGAAGAATACTCTGCTGCAGCCCGCAAGAAGAGAGGACCACATGATTAT-----------

------------------------------------------------------------------------------------------------------------TGAAGCCGCCCGATGGTATAAATAAAATCATGTGGATGCA^TTTTGCGCCGAGTGGACACTAGAGTAGGGGGACAAATCGGTACCCATTCCAGCAAAAAGTCGAAGAATACTCTGCTGCAGCCCGCAAGAAGAGAGGACCACATGATTATC----------

-------------------------------------------------------------------------------------------------------------GAAGCCGCCCGATGGTATAAATAAAATCATGTGGATGCA^TTTTGCGCCGAGTGGACACTAGAGTAGGGGGACAAATCGGTACCCATTCCAGCAAAAAGTCGAAGAATACTCTGCTGCAGCCCGCAAGAAGAGAGGACCACATGATTATCT---------

--------------------------------------------------------------------------------------------------------------AAGCCGCCCGATGGTATAAATAAAATCATGTGGATGCA^TTTTGCGCCGAGTGGACACTAGAGTAGGGGGACAAATCGGTACCCATTCCAGCAAAAAGTCGAAGAATACTCTGCTGCAGCCCGCAAGAAGAGAGGACCACATGATTATCTG--------

----------------------------------------------------------------------------------------------------------------GCCGCCCGATGGTATAAATAAAATCATGTGGATGCA^TTTTGCGCCGAGTGGACACTAGAGTAGGGGGACAAATCGGTACCCATTCCAGCAAAAAGTCGAAGAATACTCTGCTGCAGCCCGCAAGAAGAGAGGACCACATGATTATCTGTT------

----------------------------------------------------------------------------------------------------------------------CGATGGTATAAATAAAATCATGTGGATGCA^TTTTGCGCCGAGTGGACACTAGAGTAGGGGGACAAATCGGTACCCATTCCAGCAAAAAGTCGAAGAATACTCTGCTGCAGCCCGCAAGAAGAGAGGACCACATGATTATCTGTTGATGAG

---------------------------------------------------TGCCCCTGACTCGGCTATCGGGTAGCATTAGCAAAAGGAGATTTTCGACCCGCACACTGAAGCCGCCCGATGGTATAAATAAAATCATGTGGATGCA^TTTTGCGCCGAGTGGACACTAGAGTAGGGGGACAAATCGGTACCCATTCCAGC-------------------------------------------------------------------

---------------------------------------------------TGCCGCTGACTCGGCTATCGGGTAGCATTAGCAAAAGGAGATTTTCGACCCGCACACTGAAGCCGCCCGATGGTATAAATAAAATCATGTGGATGCA^TTTTGCGCCGAGTGGACACTAGAGTAGGGGGACAAATCGGTACCCATTCCAGC-------------------------------------------------------------------

---------------------------------------------------TGCCCCTGACTCGGCTATCGGGTAGCATTAGCAAAAGGAGATTTTCGACCCGCACACTGAAGCCGCCCGATGGTATAAATAAAATCATGTGGATGCA^TTTTGCGCCGAGTGGACACTAGAGTAGGGGGACAAATCGGTACCCATTCCAGC-------------------------------------------------------------------

-------------------------------------------------------CCTGACTCGGCTATCGGGTAGCATTAGCAAAAGGAGATTTTCGACCCGCACACTGAAGCCGCCCGATGGTATAAATAAAATCATGTGGATGCA^TTTTGCGCCGAGTGGACACTAGAGTAGGGGGACAAATCGGTACCCATTCCAGCAAAA---------------------------------------------------------------

--------------------------------------------------------CTGACTCGGCTATCGGGTAGCATTAGCAAAAGGAGATTTTCGACCCGCACACTGAAGCCGCCCGATGGTATAAATAAAATCATGTGGATGCA^TTTTGCGCCGAGTGGACACTAGAGTAGGGGGACAAATCGGTACCCATTCCAGCAAAAA--------------------------------------------------------------

---------------------------------------------------------TGACTCGGCTATCGGGTAGCATTAGCAAAAGGAGATTTTCGACCCGCACACTGAAGCCGCCCGATGGTATAAATAAAATCATGTGGATGCA^TTTTGCGCCGAGTGGACACTAGAGTAGGGGGACAAATCGGTACCCATTCCAGCAAAAAG-------------------------------------------------------------

-----------------------------------------------------------ACTCGGCTATCGGGTAGCATTAGCAAAAGGAGATTTTCGACCCGCACACTGAAGCCGCCCGATGGTATAAATAAAATCATGTGGATGCA^TTTTGCGCCGAGTGGACACTCGAGTAGGGGGACAAATCGGTACCCATTCCAGCAAAAAGTC-----------------------------------------------------------

--------------------------------------------------------------CGGCTATCGGGTAGCATTAGCAAAAGGAGATTTTCGACCCGCACACTGAAGCCGCCCGATGGTATAAATAAAATCATGTGGATGCA^TTTTGCGCCGAGTGGACACTAGAGTAGGGGGACAAATCGGTACCCATTCCAGCAAAAAGTCGAA--------------------------------------------------------

--------------------------------------------------------------CGCCTATCGGGTAGCATTAGCAAAAGGAGATTTTCGACCCGCACACTGAAGCCGCCCGATGGTATAAATAAAATCATGTGGATGCA^TTTTGCGCCGAGTGGACACTAGAGTAGGGGGACAAATCGGTACCCATTCCAGCAAAAAGTCGAA--------------------------------------------------------

-------------------------------------------------------------------ATCGGGTAGCATTAGCAAAAGGAGATTTTCGACCCGCACACTGAAGCCGCCCGATGGTATAAATAAAATCATGTGGATGCA^TTTTGCGCCGAGTGGACACTAGAGTAGGGGGACAAATCGGTACCCATTCCAGCAAAAAGTCGAAGAATA---------------------------------------------------

--------------------------------------------------------------------TCGGGTAGCATTAGCAAAAGGAGATTTTCGACCCGCACACTGAAGCCGCCCGATGGTATAAATAAAATCATGTGGATGCA^TTTTGCGCCGAGTGGACACTAGAGTAGGGGGACAAATCGGTACCCATTCCAGCAAAAAGTCGAAGAATAC--------------------------------------------------

---------------------------------------------------------------------CGGGTAGCATTAGCAAAAGGAGATTTTCGACCCGCACACTGAAGCCGCCCGATGGTATAAATAAAATCATGTGGATGCA^TTTTGCGCCGAGTGGACACTAGAGTAGGGGGACAAATCGGTACCCATTCCAGCAAAAAGTCGAAGAATACT-------------------------------------------------

--------------------------------------------------------------------------AGCATTAGCAAAAGGAGATTTTCGACCCGCACACTGAAGCCGCCCGATGGTATAAATAAAATCATGTGGATGCA^TTTTGCGCCGAGTGGACACTAGAGTAGGGGGACAAATCGGTACCCATTCCAGCAAAAAGTCGAAGAATACTCTGCT--------------------------------------------

--------------------------------------------------------------------------AGCATTAGCAAAAGGAGATTTTCGACCCGCACACTGAAGCCGCCCGATGGTATAAATAAAATCATGTGGATGCA^TTTTGCGCCGAGTGGACACTAGAGTAGGGGGACAAATCGGTACCCATTCCAGCAAAAAGTCGAAGAATACTCTGCT--------------------------------------------

-----------------------------------------------------------------------------ATTAGCAAAAGGAGATTTTCGACCCGCACACTGAAGCCGCCCGATGGTATAAATAAAATCATGTGGATGCA^TTTTGCGCCGAGTGGACACTAGAGTAGGGGGACAAATCGGTACCCATTCCAGCAAAAAGTCGAAGAATACTCTGCTGTA-----------------------------------------

-----------------------------------------------------------------------------ATTAGCAAAAGGAGATTTTCGACCCGCACACTGAAGCCGCCCGATGGTATAAATAAAATCATGTGCATGCA^TTTTGCGCCGAGTGGACACTAGAGTAGGGGGACAAATCGGTACCCATTCCAGCAAAAAGTCGAAGAATACTCTGCTGCA-----------------------------------------

------------------------------------------------------------------------------------AGAGGAGATTTTCGACCCGCACACTGAAGCCGCCCGATGGTATAAATAAAATCATGTGGATGCA^TTTTGCGCCGAGTGGACACTAGAGTAGGGGGACAAATCGGTACCCATTCCAGCAAAAAGTCGAAGAATACTCTGCTGCAGCCCGCA----------------------------------

--------------------------------------------------------------------------------------AGGAGATTTTCGACCCGCACACTGAAGCCGCCCGATGGTATAAATAAAATCATGTGGATGCA^TTTTGCGCCGAGTGGACACTAGACTAGGGGGACAAATCGGTACCCATTCCAGCAAAAAGTCGAAGAATACTCTGCTGCAGCCCGCAAG--------------------------------

---------------------------------------------------------------------------------------GGAGATTTTCGACCCGCACACTGAAGCCGCCCGATGGTATAAATAAAATCATGTGGATGCA^TTTTGCGCCGAGTGGACACTAGAGTAGGGGGACAAATCGGTACCCATTCCAGCAAAAAGTCGAAGAATACTCTGCTGCAGCCCGCAAGA-------------------------------

----------------------------------------------------------------------------------------GAGATTTTCGACCCGCACACTGAAGCCGCCCGATGGTATAAATAAAATCATGTGGATGCA^TTTTGCGCCGAGTGGACACTAGAGTAGGGGGACAAATCGGTACCCATTCCAGCAAAAAGTCGAAGAATACTCTGCTGCAGCCCGCAAGAA------------------------------

---------------------------------------------------------------------------------------------TTTCGACCCGCACACTGAAGCCGCCCGATGGTATAAATAAAATCATGTGGATGCA^TTTTGCGCCGAGTGGACACTAGAGTAGGGGGACAAATCGGTACCCATTCCAGCAAAAAGTCGAAGAATACTTTGCTGCAGCCCGCAAGAAGAGAC-------------------------

----------------------------------------------------------------------------------------------TTCGACCCGCACACTGAAGCCGCCCGATGGTATAAATAAAATCATGTGGATGCA^TTTTGCGCCGAGTGGACACTAGAGTAGGGGGACAAATCGGTACCAATTCCAGCAAAAAGTCGAAGAATACTCTGCTGCAGCCCGCAAGAAGAGAGG------------------------

-----------------------------------------------------------------------------------------------TCGACCCGCACACTGAAGCCGCCCGATGGTATAAATAAAATCATGTGGATGCA^TTTTGCGCCGAGTGGACACTAGAGTAGGGGGACAAATCGGTACCCATTCCAGCAAAAAGTCGAAGAATACTCTGCTGCAGCCCGCAAGAAGAGAGGA-----------------------

------------------------------------------------------------------------------------------------CGACGCGCACACTGAAGCCGCCCGATGGTATAAATAAAATCATGTGGATGCA^TTTTGCGCCGAGTGGACACTAGAGTAGGGGGACAAATCGGTACCCATTCCAGCAAAAAGTCGAAGAATACTCTGCTGCAGCCCGCAAGAAGATAGGAC----------------------

-------------------------------------------------------------------------------------------------GACCCGCACACTGAAGCCGCCCGATGGTATAAATAAAATCATGTGGATGCA^TTTTGCGCCGAGTGGACACTAGAGTAGGGGGACAAATCGGTACCCATTCCAGCAAAAAGTCGAAGAATACTCTGCTGCAGCCCGCAAGAAGAGAGGACC---------------------

-------------------------------------------------------------------------------------------------GACCCGCACACTGAAGCCGCCCGATGGTATAAATAAAATCATGTGGATGCA^TTTTGCGCCGAGTGGACACTAGAGTAGGGGGACAAATCGGTACCCATTCCAGCAAAAAGTCGAAGAATACTCTGCTGCAGCCCGCAAGAAGAGAGGACC---------------------

ATAATATCCCGAACGACCGCTGGCCCTGGACGGAAGCTAATGGACGGGCGCTGCCCCTGACTCGGCTATCGGGTAGCATTAGCAAAAGGAGATTTTCGACCCGCACACTGAAGCCGCCCGATGGTATAAATAAAATCATGTGGATGCA^TT----------------------------------------------------------------------------------------------------------------------

---ATATCCCGAACGACCGCTGGCCCTGGACGGAAGCTAATGGACGGGCGCTGCCCCTGACTCGGCTATCGGGTAGCATTAGCAAAAGGAGATTTTCGACCCGCACACTGAAGCCGCCCGATGGTATAAATAAAATCATGTGGATGCA^TTTTG-------------------------------------------------------------------------------------------------------------------

------TCCCGAACGACCGCTGGCCCTGGACGGAAGCTAATGGACGGGCGCTGCCCCTGACTCGGCTATCGGGTAGCATTAGCAAAAGGAGATTTTCGACCCGCACACTGAAGCCGCCCGATGGTATAAATAAAATCATGTGGATGCA^TTTTGCGC----------------------------------------------------------------------------------------------------------------

-------CCCGAACGACCGCTGGCCCTGGACGGAAGCTAATGGACGGGCGCTGCCCCTGACTCGGCTATCGGGTAGCATTAGCAAAAGGAGATTTTCGACCCGCACACTGAAGCCGCCCGATGGTATAAATAAAATCATGTGGATGCA^TTTTGCGCC---------------------------------------------------------------------------------------------------------------

--------------GACCGCTGGCCCTGGACGGAAGCTAATGGACGGGCGCTGCCCCTGACTCGGCTATCGGGTAGCATTAGCAAAAGGAGATTTTCGACCCGCACACTGAAGCCGCCCGATGGTATAAATAAAATCATGTGGATGCA^TTTTGCGCCGAGTGGA--------------------------------------------------------------------------------------------------------

--------------------TGGCCCTGGACGGAATCTAATGGACGGGCGCTGCCCCTGACTCGGCTATCGGGTAGCATTAGCAAAAGGAGATTTTCGACCCGCACACTGAAGCCGCCCGATGGTATAAATAAAATCATGTGGATGCA^TTTTGCGCCGAGTGGACACTAG--------------------------------------------------------------------------------------------------

--------------------TGGCCCTGGACGGAAGCTAATGGACGGGCGCTGCCCCTGACTCGGCTATCGGGTAGCATTAGCAAAAGGAGATTTTCGACCCGCACACTGAAGCCGCCCGATGGTATAAATAAAATCATGTGGATGCA^TTTTGCGCCGAGTGGACACTAG--------------------------------------------------------------------------------------------------

---------------------------------AAGCTAATGGACGGGCGCTGCCCCTGACACGGCTATCGGGTAGCATTAGCAAAAGGAGATTTTCGACCCGCACACTGAAGCCGCCCGATGGTACAAATAAAATCATGTGGATGCA^TTTTGCGCCGAGTGGACACTAGAGTAGGGGGACAA-------------------------------------------------------------------------------------

------------------------------------CTAATGGACGGGCGCTGCCCCTGACTCGGCTATCGGGTAGCATTAGCAAAAGGAGATTTTCGACCCGCACACTGAAGCCGCCCGATGGTATAAATAAAATCATGTGGATGCA^TTTTGCGCCGAGTGGACACTAGAGTAGGGGGACAAATC----------------------------------------------------------------------------------

---------------------------------------ATGGACGGGCGCTGCCCCTGACTCGGCTATCGGGTAGCATTAGCAAAAGGAGATTTTCGACCCGCACACTGAAGCCGCCCGATGGTATAAATAAAATCATGTGGATGCA^TTTTGCGCCGAGTGGACACTAGAGTAGGGGGACAAATCGGT-------------------------------------------------------------------------------

----------------------------------------TGGACGGGCGCTGCCCCTGACTCGGCTATCGGGTAGCATTAGCAAAAGGAGATTTTCGACCCGCACACTGAAGCCGCCCGATGGTATAAATAAAATCATGTGGATGCA^TTTTGCGCCGAGTGGACACTAGAGTAGGGGGACAAATCGGTA------------------------------------------------------------------------------

**---ATATCCCGAACGACCGCTGGCCCTGGACGGAAGCTAATGGACGGGCGCTGCCCCTGACTCGGCTATCGGGTAGCATTAGCAAAAGGAGATTTTCGACCCGCACACTGAAGCCGCCCGATGGTATAAATAAAATCATGTGGATGCA^TTTTGCGCCGAGTGGACACTAGAGTAGGGGGACAAATCGGTACCCATTCCAGCAAAAAGTCGAAGAATACTCTGCTGCAGCCCGCAAGAAGAGAGGACCACATGATTATCTGTT------**

>R2_Flank_Consensus

TTTTGCGCCGAGTGGACACTAGAGTAGGGGGACAAATCGGTACCCATTCCAGCAAAAAGTCGAAGAATACTCTGCTGCAGCCCGCAAGAAGAGAGGACCACATGATTATCTGTT

**S13 Data. Chromosomal localization of insertions via flanking sequence alignment.**

FASTA-format alignments of consensus flanking sequences (shown in bold in S12 Data) mapped to the simulated 1 Mb host reference genome. Four distinct flanking sequences resolve to two independent chromosomal insertions through co-localization analysis. L1 and R1 map to immediately adjacent positions at chromosome coordinate ~746,864, while L2 and R2 map to immediately adjacent positions at coordinate ~500,001. The immediately adjacent mapping of left and right flanking sequences at each locus enables unambiguous junction pairing. Co-localization distinguishes L1+R1 as one insertion (containing construct bases 263-3,194; 2,932 bp) and L2+R2 as an independent insertion (containing construct bases 280-3,416; 3,137 bp) at distinct genomic loci separated by approximately 247 kb.

| **Query ID** | **Subject ID** | **% Identity** | **Alignment Length** | **Mismatches** | **Gap Openings** | **Query Start** | **Query End** | **Subject Start** | **Subject End** | **E-Value** | **Bit Score** |
| --- | --- | --- | --- | --- | --- | --- | --- | --- | --- | --- | --- |
| **L1_Flank_Consensus** | genome | 100.00 | 118 | 0 | 0 | 1 | 118 | 746746 | 746863 | 8.4e-48 | 183.2 |
| **L2_Flank_Consensus** | genome | 100.00 | 115 | 0 | 0 | 1 | 115 | 499886 | 500000 | 7e-45 | 173.5 |
| **R1_Flank_Consensus** | genome | 100.00 | 111 | 0 | 0 | 1 | 111 | 746864 | 746974 | 7.4e-42 | 163.4 |
| **R2_Flank_Consensus** | genome | 100.00 | 114 | 0 | 0 | 1 | 114 | 500001 | 500114 | 1.6e-43 | 168.9 |

```
# fasta36 -Xo -E "1e-20 -1" flank_consensus_sequences.fasta genome.fa
FASTA searches a protein or DNA sequence data bank
 version 36.3.8i Aug, 2024
Please cite:
 W.R. Pearson & D.J. Lipman PNAS (1988) 85:2444-2448

Query: flank_consensus_sequences.fasta
  1>>>L1_Flank_Consensus - 118 nt
Library: genome.fa
  1000000 residues in     1 sequences

Statistics: (shuffled [500]) MLE statistics: Lambda= 0.1978;  K=0.01896
 statistics sampled from 7 (7) to 497 sequences
Algorithm: FASTA (3.8 Nov 2011)
Parameters: +5/-4 matrix (5:-4), open/ext: -12/-4
 ktup: 6, E-join: 0.25 (0.143), E-opt: 0.05 (0), width:  16
 Scan time:  3.390

The best scores are:                                      opt bits E(7)
genome                                         (1000000) [f]  590 177.5 4.3e-46

>>genome                                                  (1000000 nt)
 initn: 590 init1: 590 opt: 590  Z-score: 874.8  bits: 177.5 E(7): 4.3e-46
banded Smith-Waterman score: 590; 100.0% identity (100.0% similar) in 118 nt overlap (1-118:746746-746863)

                                             10        20        30
L1_Fla                               TCAGCGTCAAGGCCGACTGCTATACATAAA
                                     ::::::::::::::::::::::::::::::
genome GCCGATAGCTAGCTGTCGTACTGAAAGAAATCAGCGTCAAGGCCGACTGCTATACATAAA
      746720    746730    746740    746750    746760    746770

               40        50        60        70        80        90
L1_Fla ACCACGTTGGTGATAGACCGTTTTGGCAGTCCACCTTTTGTTCACGAAGTAGCTATGGTA
       ::::::::::::::::::::::::::::::::::::::::::::::::::::::::::::
genome ACCACGTTGGTGATAGACCGTTTTGGCAGTCCACCTTTTGTTCACGAAGTAGCTATGGTA
      746780    746790    746800    746810    746820    746830

              100       110
L1_Fla AGCTACTGCGGTGTATTATCCCAAAGCT
       ::::::::::::::::::::::::::::
genome AGCTACTGCGGTGTATTATCCCAAAGCTACCACGCGGGTGTTTATATCCCCAATGGGACA
      746840    746850    746860    746870    746880    746890


  2>>>L2_Flank_Consensus - 115 nt
Library: genome.fa
  1000000 residues in     1 sequences

Statistics: (shuffled [500]) MLE statistics: Lambda= 0.2042;  K=0.03122
 statistics sampled from 7 (7) to 500 sequences
Algorithm: FASTA (3.8 Nov 2011)
Parameters: +5/-4 matrix (5:-4), open/ext: -12/-4
 ktup: 6, E-join: 0.25 (0.179), E-opt: 0.05 (0), width:  16
 Scan time:  1.920

The best scores are:                                      opt bits E(7)
genome                                         (1000000) [f]  575 177.9 3.3e-46

>>genome                                                  (1000000 nt)
 initn: 575 init1: 575 opt: 575  Z-score: 876.8  bits: 177.9 E(7): 3.3e-46
banded Smith-Waterman score: 575; 100.0% identity (100.0% similar) in 115 nt overlap (1-115:499886-500000)

                                             10        20        30
L2_Fla                               GATACCATCCTTCGATTCGTGGCTGTAGAT
                                     ::::::::::::::::::::::::::::::
genome TCGGATGTGCAAGCGTCAGCCCTGTTAAAAGATACCATCCTTCGATTCGTGGCTGTAGAT
      499860    499870    499880    499890    499900    499910

               40        50        60        70        80        90
L2_Fla AGATCTTAAATAGTTCACTTATACCTTGGATTTAACGGCCGATTCTAAAACACTTCGAGG
       ::::::::::::::::::::::::::::::::::::::::::::::::::::::::::::
genome AGATCTTAAATAGTTCACTTATACCTTGGATTTAACGGCCGATTCTAAAACACTTCGAGG
      499920    499930    499940    499950    499960    499970

              100       110
L2_Fla GCTAGTCAAGGAGGCCATACTAACC
       :::::::::::::::::::::::::
genome GCTAGTCAAGGAGGCCATACTAACCTTTTGCGCCGAGTGGACACTAGAGTAGGGGGACAA
      499980    499990    500000    500010    500020    500030


3>>>R1_Flank_Consensus - 111 nt
Library: genome.fa
  1000000 residues in     1 sequences

Statistics: (shuffled [500]) MLE statistics: Lambda= 0.1990;  K=0.02158
 statistics sampled from 7 (7) to 499 sequences
Algorithm: FASTA (3.8 Nov 2011)
Parameters: +5/-4 matrix (5:-4), open/ext: -12/-4
 ktup: 6, E-join: 0.25 (0.167), E-opt: 0.05 (0), width:  16
 Scan time:  2.080

The best scores are:                                      opt bits E(7)
genome                                         (1000000) [f]  555 168.3 2.5e-43

>>genome                                                  (1000000 nt)
 initn: 555 init1: 555 opt: 555  Z-score: 825.3  bits: 168.3 E(7): 2.5e-43
banded Smith-Waterman score: 555; 100.0% identity (100.0% similar) in 111 nt overlap (1-111:746864-746974)

                                             10        20        30
R1_Fla                               ACCACGCGGGTGTTTATATCCCCAATGGGA
                                     ::::::::::::::::::::::::::::::
genome TAAGCTACTGCGGTGTATTATCCCAAAGCTACCACGCGGGTGTTTATATCCCCAATGGGA
        746840    746850    746860    746870    746880    746890

               40        50        60        70        80        90
R1_Fla CAGGCTCCCACGTTCGGCTATTGACTCAGAAAATTGACAGCTGAGTGCGCATAATCATAG
       ::::::::::::::::::::::::::::::::::::::::::::::::::::::::::::
genome CAGGCTCCCACGTTCGGCTATTGACTCAGAAAATTGACAGCTGAGTGCGCATAATCATAG
        746900    746910    746920    746930    746940    746950

              100       110
R1_Fla ATAGGCACCTACAGTCAACAA
       :::::::::::::::::::::
genome ATAGGCACCTACAGTCAACAACTCCATACAATGCTTTTGCAAGTTGCTTGCCCAAGATCG
        746960    746970    746980    746990    747000    747010


  4>>>R2_Flank_Consensus - 114 nt
Library: genome.fa
  1000000 residues in     1 sequences

Statistics: (shuffled [500]) MLE statistics: Lambda= 0.1874;  K=0.01028
 statistics sampled from 7 (7) to 499 sequences
Algorithm: FASTA (3.8 Nov 2011)
Parameters: +5/-4 matrix (5:-4), open/ext: -12/-4
 ktup: 6, E-join: 0.25 (0.161), E-opt: 0.05 (0), width:  16
 Scan time:  1.890

The best scores are:                                      opt bits E(7)
genome                                         (1000000) [f]  570 164.1 4.6e-42

>>genome                                                  (1000000 nt)
 initn: 570 init1: 570 opt: 570  Z-score: 802.4  bits: 164.1 E(7): 4.6e-42
banded Smith-Waterman score: 570; 100.0% identity (100.0% similar) in 114 nt overlap (1-114:500001-500114)

                                             10        20        30
R2_Fla                               TTTTGCGCCGAGTGGACACTAGAGTAGGGG
                                     ::::::::::::::::::::::::::::::
genome CGAGGGCTAGTCAAGGAGGCCATACTAACCTTTTGCGCCGAGTGGACACTAGAGTAGGGG
           499980    499990    500000    500010    500020    500030

               40        50        60        70        80        90
R2_Fla GACAAATCGGTACCCATTCCAGCAAAAAGTCGAAGAATACTCTGCTGCAGCCCGCAAGAA
       ::::::::::::::::::::::::::::::::::::::::::::::::::::::::::::
genome GACAAATCGGTACCCATTCCAGCAAAAAGTCGAAGAATACTCTGCTGCAGCCCGCAAGAA
           500040    500050    500060    500070    500080    500090

              100       110
R2_Fla GAGAGGACCACATGATTATCTGTT
       ::::::::::::::::::::::::
genome GAGAGGACCACATGATTATCTGTTGATGAGAAATCAGCAAGAAGTCGCCTAGCTCCGGTC
           500100    500110    500120    500130    500140    500150


458 residues in 4 query   sequences
1000000 residues in 1 library sequences
 Tcomplib [36.3.8i Aug, 2024] (10 proc in memory [0G])
 start: Wed Dec 17 10:35:40 2025 done: Wed Dec 17 10:35:41 2025
 Total Scan time:  9.280 Total Display time:  0.010

Function used was FASTA [36.3.8i Aug, 2024]
```
